# Discovery of RNA-binding fragments using biolayer interferometry

**DOI:** 10.1039/d5md00673b

**Published:** 2025-09-19

**Authors:** Vipul Navinchandra Panchal, Jan-Åke Husmann, Kaja Günther, Muhammad Zeeshan, Bengt Erik Haug, Ruth Brenk

**Affiliations:** a Department of Biomedicine, University of Bergen Jonas Lies vei 91 5020 Bergen Norway ruth.brenk@uib.no; b Department of Chemistry and Centre for Pharmacy, University of Bergen Allégaten 41 5007 Bergen Norway; c Computational Biology Unit, University of Bergen Thormøhlensgate 55 5008 Bergen Norway

## Abstract

Structured RNAs are increasingly explored as novel pharmacological targets for a range of diseases. Therefore, evaluating methods for RNA-focused hit discovery is crucial. Biolayer Interferometry (BLI), a label-free technique that detects biomolecular interactions by measuring changes in white light interference near the sensor surface, offers high throughput and multiplexing capabilities. While BLI has been widely adopted for protein-targeted screening, its application in RNA-targeted drug discovery remains largely unexplored. In this study, we demonstrate the effective use of BLI to investigate RNA–small molecule interactions using three different riboswitches, which are potential targets for novel antibiotics. Furthermore, we describe the successful use of BLI to identify fragment binders of these RNA targets. We combined the BLI experiments with ligand-based NMR as an orthogonal validation method and were able to identify seven competitive fragment binders of the flavin mononucleotide (FMN) riboswitch, each featuring scaffolds distinct from the previously known ligands.

## Abstract 2

A combinatorial approach integrating BLI and ligand-based NMR for RNA-focused fragment-based drug discovery.

## Graphical abstract

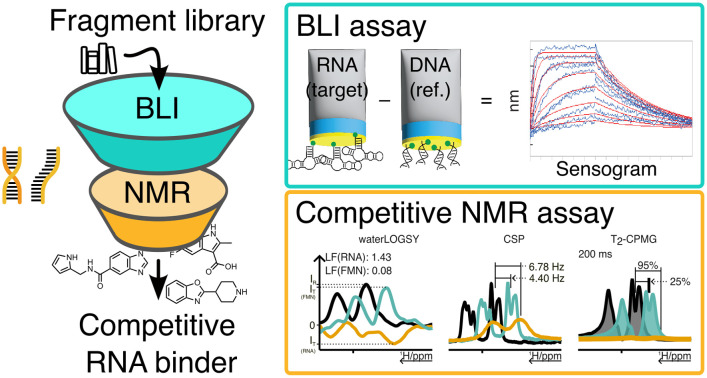

## Introduction

Regulatory elements within mRNAs across different life forms are increasing being recognised to modulate diverse biological processes.^1,2^ Several studies have revealed association of pathological conditions with the dysregulation of RNA elements.^3–5^ The ability of RNA to fold into complex three-dimensional structures that form well-defined pockets-sophisticated enough to selectively bind small molecules, provides a unique opportunity to target difficult-to-drug proteins and thereby potentially expanding the current druggable genome.^6–8^ Consequently, RNAs have recently emerged as attractive pharmacological targets for various pathological conditions including infectious diseases.^9–12^

Fragment-based drug discovery (FBDD) enables efficient exploration of chemical space, often beyond what larger molecules can achieve,^13–15^ and has delivered several drugs targeting proteins.^13^ However, FBDD approaches are underexplored for RNA targets.^16^ A key challenge in fragment-based approaches targeting RNA is the limited number of effective techniques available for identifying RNA-binding fragment ligands. To date FBDD approaches against RNA targets include RNA and ligand observed NMR spectroscopy,^17–19^ competition assays using a labelled ligand,^20,21^ and chemical probing techniques.^22,23^ However, these techniques are either low-medium throughput, require medium-large amounts of RNA samples, or involve complex multi-step data analysis.

Biolayer interferometry (BLI) is a real-time, label-free technique that runs on a plate-based dip-and-read platform.^24^ It measures changes in the biolayer thickness caused by ligand binding to an immobilised binding partner on the BLI biosensor surface in form of interference patterns. Due to the low sample requirement (approx. 50–200 pmol), and the capacity to simultaneously read from 8–16 interactions (depending on the BLI platform), BLI is a powerful screening technique with good throughput. Indeed, BLI has been used successfully against protein targets in fragment screening campaigns.^25–27^ To our knowledge, up to now only two studies have employed biolayer interferometry (BLI) to quantify the binding affinities of small-molecule ligands (between 300–600 Da) against RNA targets of 30–50 nucleotides.^28,29^

**Fig. 1:**
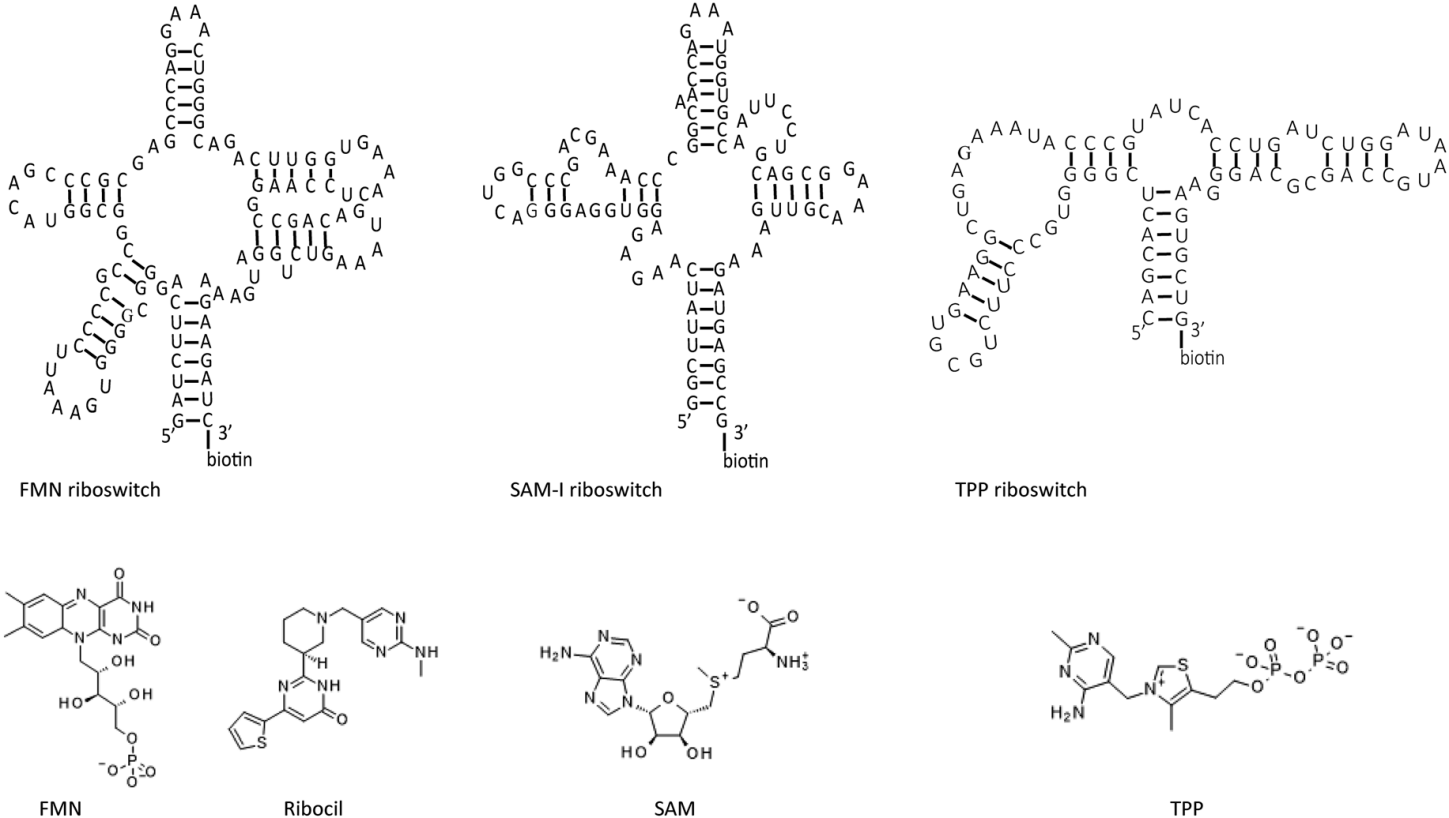
Different riboswitches and their high affinity ligands. From left to right, sequences of FMN, SAM-I and TPP riboswitch used in the study shown as 2D structures. Below each riboswitch their respective high affinity ligands, namely FMN, ribocil, SAM and TPP are displayed.

Here, we demonstrate the effective use of BLI to investigate RNA–small molecule interactions together with a competitive NMR assay. To thoroughly evaluate the suitability of BLI to characterize RNA–small molecule interactions and to identify RNA binding fragments, we focused on three different riboswitches, namely the flavin mononucleotide (FMN), *S*-adenosyl-methionine-I (SAM-I) and thiamine pyrophosphate (TPP) riboswitches (Fig. 1). Riboswitches are structured non-coding mRNA elements primarily found in bacteria which regulate the expression of their own mRNA by binding small molecules or ions, making them promising targets for new antibiotics and therefore highly relevant to fight the global rise in antibiotic resistance.^9,30,31^ For example, in its phosphorylated form, roseoflavin, a natural riboflavin analogue, inhibits the growth of *B. subtilis*, *E. coli*, and *L. monocytogenes* by targeting the FMN riboswitch.^32–34^ Similarly, pyrithiamine, originally designed as a structural analogue of thiamine to study thiamine metabolism, is toxic to bacteria and was found to target the TPP riboswitch.^35,36^ Currently, many compounds targeting riboswitches are structural analogues of their natural ligands, rendering the compounds difficult to modify and potentially leading to off-target effects and toxicity.^9,37^ However, the discovery of ribocil (Fig. 1), a flavin mononucleotide (FMN) riboswitch targeting antibiotic, demonstrated that riboswitches can bind ligands with distinct chemical scaffolds,^38^ setting precedence for designing selective drug-like molecules which are less likely to interfere with host metabolism.

By probing interactions between established riboswitch targets and their cognate ligands (Fig. 1), we demonstrate Mg^2+^ dependent changes in the BLI response. Subsequently, the optimized conditions were used to screen a fragment library against the FMN and TPP riboswitch. Primary BLI hits were further confirmed through a dose–response assay. Hits were subsequently evaluated using an orthogonal NMR spectroscopy assay to determine whether they bind competitively with the cognate riboswitch ligands. This was done using a combination of waterLOGSY, chemical shift perturbation (CSP), and *T*_2_ relaxation decay experiments. This combined approach led to the discovery of seven novel, competitive FMN riboswitch binding fragments with affinities in the 14–500 μM range.

## Results and discussion

**Fig. 2:**
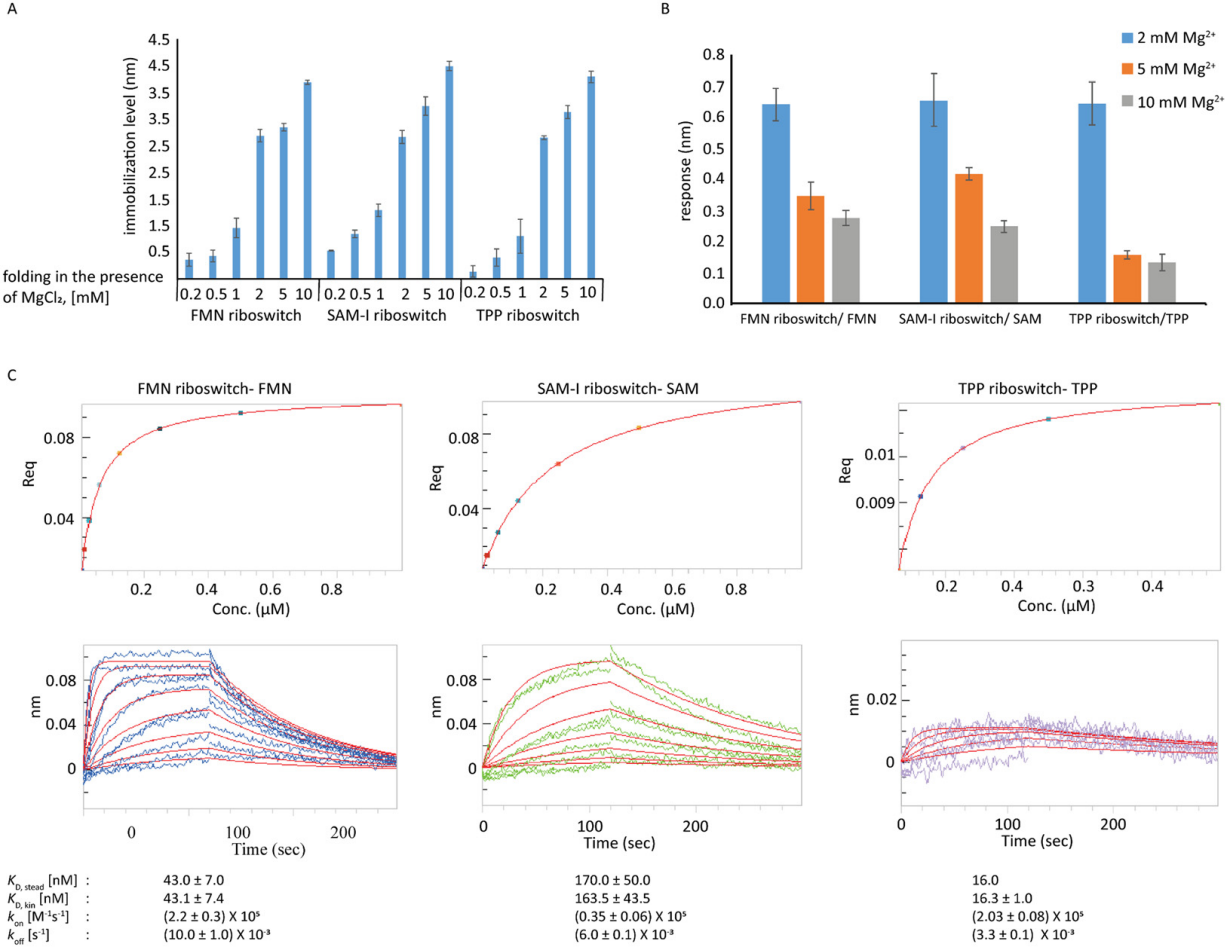
Effect of Mg^2+^ concentration on A) riboswitch immobilization and B) interaction with the cognate ligands. Representative sensograms of each riboswitch–ligand pair at each Mg^2+^ concentration are shown in Fig. S1. C) The steady state models (upper graphs) and kinetic fit (1 : 1) model (bottom graphs) for the FMN riboswitch–FMN, SAM-I riboswitch–SAM and TPP riboswitch–TPP pairs are displayed. The fitting curve is shown in red. For each parameter, the average of two independent measurements together with standard deviation are shown, except for the TPP riboswitch–TPP pair for which the data are only from a single measurement.

The tertiary structure of riboswitches plays a pivotal role in regulating gene expression upon the binding of its cognate ligand.^39,40^ The structural organization of RNA elements, which involves the folding of secondary structure elements into a stable tertiary structure, is highly dependent on Mg^2+^ and often crucial for obtaining a ligand-binding conformation. In addition, changing the salt concentration can help to minimize unspecific binding to the sensor surface. Therefore, we first evaluated how varying the MgCl_2_ concentration during RNA folding of the investigated riboswitches influences their immobilization on super streptavidin (SSA) biosensors and subsequent binding to their respective cognate ligands. For that purpose, the different riboswitches were biotinylated at the 3′ end, refolded in increasing concentrations of MgCl_2_, and loaded to SSA sensors. The loading data shows that an increasing MgCl_2_ concentration during the folding process led to enhanced immobilization levels for all three riboswitches (Fig. 2A). Notably, a >4-fold increase in immobilization levels was observed when using 2 mM MgCl_2_ for refolding compared to 0.2 mM MgCl_2_, while the highest immobilization levels of about 3.7 nM were achieved at the highest tested concentration of 10 mM MgCl_2_. Therefore, for all subsequent experiments, the immobilization was carried out using riboswitches refolded in the presence of 10 mM MgCl_2_.

Next, the performance of the BLI dip and read biosensors for detecting ligand binding was evaluated by probing the interactions of the three riboswitches against their respective cognate ligands, FMN, TPP, and SAM, at 5 μM in a Mg^2+^ dependent manner. Interestingly, we observed a Mg^2+^-dependent decrease in the response for all three pairs: the highest response was observed for the interaction in the presence of 2 mM MgCl_2_ and the lowest for the interaction in the presence of 10 mM MgCl_2_ (Fig. 2B and S1). This trend is opposite to that observed for RNA loading, and its underlying cause remains unclear. Further investigation of this phenomenon was beyond the scope of the present study.

Based on these results, we further characterized these interactions in the presence of 2 mM MgCl_2_ in a serial dilution series ranging from 7.8–1000 nM for FMN and SAM, and 3.9–500 nM for TPP. Affinities from steady state and kinetic fit (1 : 1) models were in good agreement with each other (Fig. 2C). The estimated binding affinities based on the steady state model were 43 nM for the FMN riboswitch/FMN pair, 170 nM for the SAM-I riboswitch/SAM pair and 16 nM for the TPP riboswitch/TPP pair. These values are close to the previously reported values of approximately 10 nM, 190 nM, and 50 nM, respectively.^41–43^ Compared to the other pairs, the TPP riboswitch/TPP pair resulted in responses with a low signal-to-noise ratio which could not be improved with repeated attempts. The reasons for this remained unclear. Nevertheless, these data established conditions suitable to study riboswitch–small molecule interactions using BLI by i) immobilization of riboswitch ligands folded in the presence of 10 mM MgCl_2_ and ii) measurements of interactions in the presence of 2 mM MgCl_2_.

**Fig. 3:**
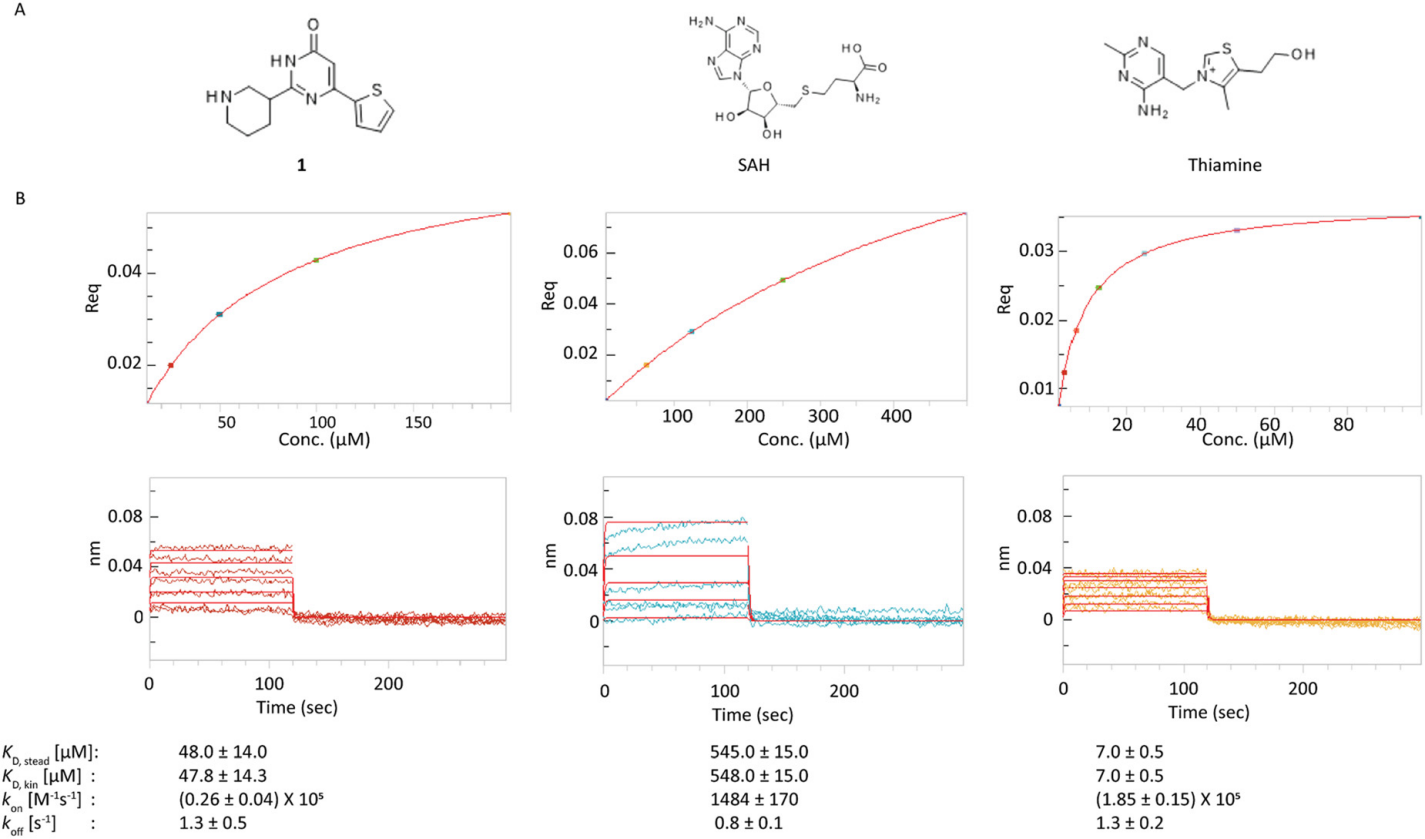
Profile of interaction between riboswitch and weak ligands. A) Structures of ligands investigated. B) Steady state models (upper graphs) and kinetic fit (1 : 1) model (bottom graphs) for the FMN riboswitch–1, SAM-I riboswitch–SAH and TPP riboswitch–thiamine pairs with calculated kinetic parameters. For each parameter, average of two independent measurements together with standard deviation are shown.

As we aimed to evaluate BLI as a primary method to identify fragment binders, we continued with testing binding of weak ligands. For this purpose, we synthesized compound 1, a fragment of the potent ribocil FMN ligand, while for the SAM-I and TPP riboswitch, *S*-adenosyl-l-homocysteine (SAH) and thiamine were used, respectively, as they bind with tens-of-micromolar affinity (Fig. 3A).^41,42^ These interactions were characterized in a serial dilution series ranging from 1.56–200 μM for 1, 7.8–500 μM for SAH, and 0.78–100 μM for thiamine in the presence of 2 mM MgCl_2_. The binding affinities of the FMN, SAM-I, and TPP riboswitches for the investigated weak ligands based on both steady-state and kinetic fit (1 : 1) models were in good agreement with each other. The estimated binding affinities based on the steady state model were 48 μM, 545 μM, and 7.0 μM for the FMN riboswitch/1, SAM-I riboswitch/SAH, and TPP riboswitch/thiamine pairs, respectively (Fig. 3B). As expected, the affinity of 1 was much lower than that of the ribocil A (16 nM).^38^ The affinities of SAH and thiamine were roughly in agreement with the previously reported values: 71 μM and 50 μM, respectively.^41,42^ Here, the signal-to-noise ratio for the TPP riboswitch/thiamine pair was comparable to that of the FMN riboswitch/1 pair which could suggest that the failure to obtain good data with TPP is due to issues with the compound and not the analytical method.

**Fig. 4:**
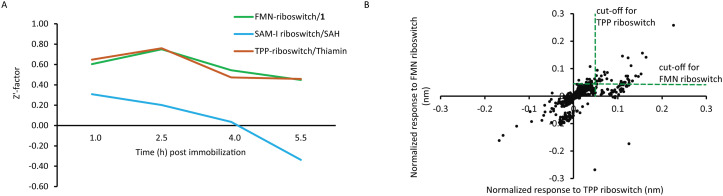
BLI enabled fragment screening. A) The *Z*′-factor over 10 replicates of three riboswitch targets and their weak ligands at 100 μM as a function of time for the optimised BLI assay. B) Scatter plot with the responses of the individual fragments from the fragment library against the FMN (*y*-axis) and TPP riboswitch (*x*-axis). The cut-offs used to select fragments for further characterization (0.42 nm for the FMN and 0.46 for the TPP riboswitch) are shown as green dotted lines.

To assess if the binding assay is suitable for fragment screening, we further studied the assay quality for these riboswitch targets over at 90 min intervals using *Z*′-factor analysis. The *Z*′-factor is often used to judge whether the response in a particular assay is large enough to warrant further attention.^44^ One hour after immobilization, the *Z*′-factors for the FMN riboswitch–1, SAM-I riboswitch–SAH, and TPP riboswitch–thiamine pairs were 0.60, 0.31, and 0.65, respectively. After approximately 5.5 h post-immobilisation, the *Z*′-factors declined to 0.45, −0.34, and 0.46 (Fig. 4A). Considering that *Z*′-factors above 0.5 represent an excellent screening assay and values between 0 to 0.5 still represent a marginal assay,^44^ the BLI assay clearly performed within the acceptable range up to 5.5 h to reliably identify potential fragment binders for FMN and TPP riboswitch allowing to use just one set of sensors to screen up to three plates on the same day. Due to the poor performance of the SAM-I–riboswitch–SAH pair, it was excluded in the subsequent fragment screening campaign.

Next, an in-house fragment library comprising 651 fragments was screened in sets of 80 fragments per plate against the FMN and TPP riboswitches.^26^ Each plate was read by three sets of sensors sequentially: first eight sensors immobilized with dsDNA as a reference, second FMN riboswitch immobilized sensors and third TPP riboswitch immobilized sensors. Control measurements were included at the end of each screening plate to monitor the residual activity of the riboswitches. Fig. 4B shows the range of responses of fragments against the two riboswitch targets. A threshold of >1.0 sigma over the median response was used to select potential binders while at the same time those with an unusually high response were rejected. Using this approach, the screening identified 35 fragment binders (5.4% hit rate) which were selected for subsequent hit validation. Of the 35 hits, two were unique to the FMN riboswitch (termed “FMN riboswitch hits”), and six were unique to the TPP riboswitch-termed (“TPP riboswitch hits”), whereas the remaining 27 fragments showed responses to both riboswitches (“common hits”) (Table S1). All 35 fragments were further evaluated in a serial dilution series ranging from 1–200 μM against both the FMN and TPP riboswitches. Against the FMN riboswitch, 30 fragments showed a dose-dependent response (Fig. S2). Interestingly, six of them were from the TPP riboswitch hit list, one from the FMN riboswitch hit list, and the remaining ones were among the common hits. Against the TPP riboswitch, only fragment 31 from the common hit list showed a dose-dependent increase in the response (Fig. S3).

Based on the visual inspection of the dose–response curves, we selected 21 fragments for further evaluation: 20 FMN riboswitch hits and fragment 31 that gave dose response against both riboswitches. For the nine fragments that were rejected, less than 4 dose–response data points above the threshold response of 0.005 nm in the investigated concentration range could be measured (Table S1).

**Fig. 5:**
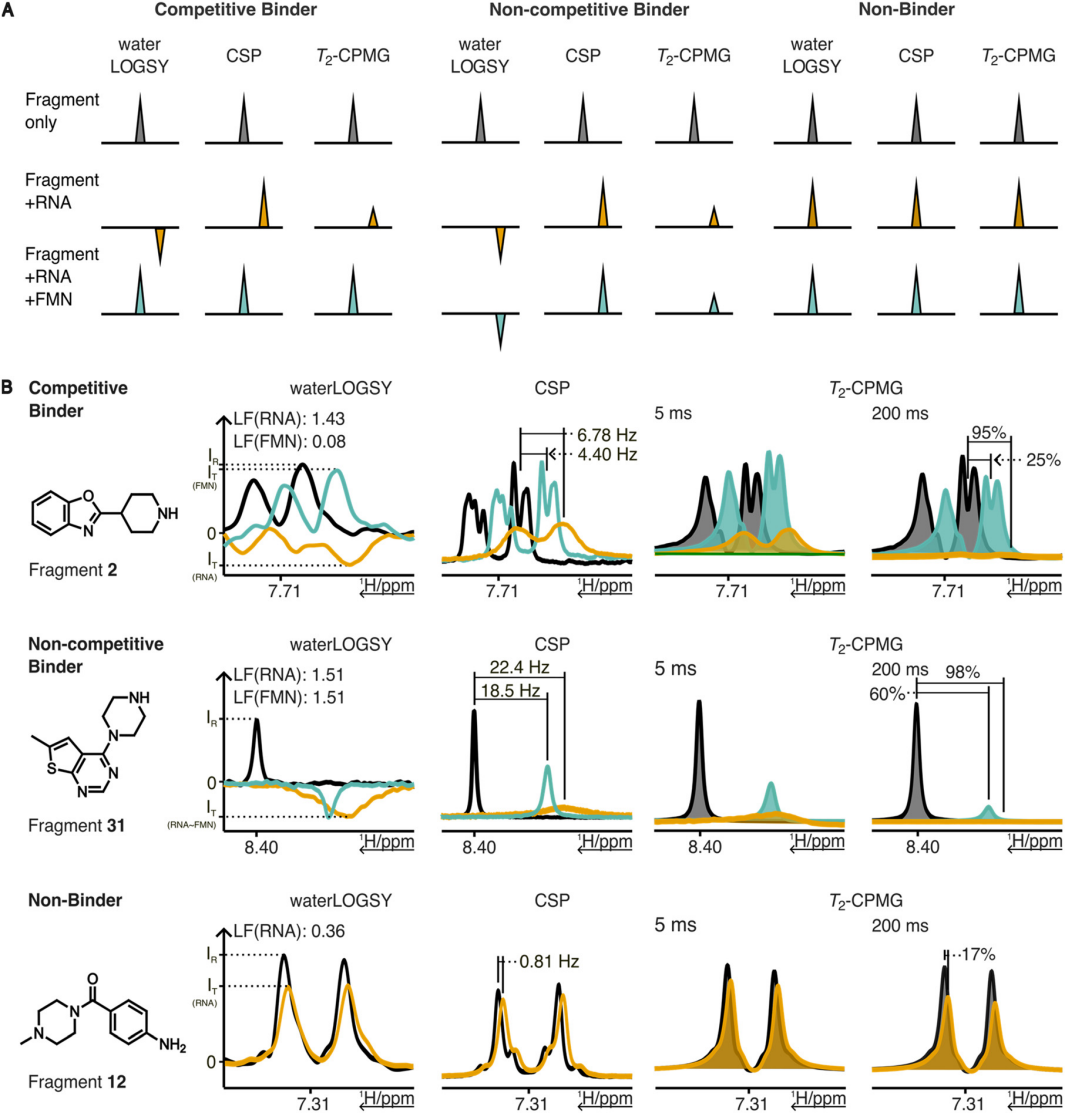
NMR-based assay for binding site validation. A) Schematic representation of NMR experiments and criteria used to distinguish between competitive and non-competitive binders as well as non-binders based on results from waterLOGSY, chemical shift perturbation (CSP), and *T*_2_ relaxation decay (*T*_2_-CPMG) NMR experiments. Colour coding: fragment only (black), fragment in presence of RNA (orange), and fragment in presence of RNA and competitor FMN, TPP, or SAM (cyan). B) Representative NMR results obtained for a competitive binder, non-competitive binder as well as a non-binder among the fragments identified in the BLI screen. Fragments exceeding the thresholds (LOGSY factor (LF(RNA)) > 1 as well as CSP > 6 Hz and/or *T*_2_ reduction >60%) upon RNA addition were classified as binder. Upon the addition of a strong binder (FMN) a shift towards the reference signals (signals below the threshold) indicated competitive binding (top), while non-competitive binder remained above the threshold (middle). Fragments remaining under the threshold in the presence of RNA were classified as non-binder (bottom).

**Fig. 6:**
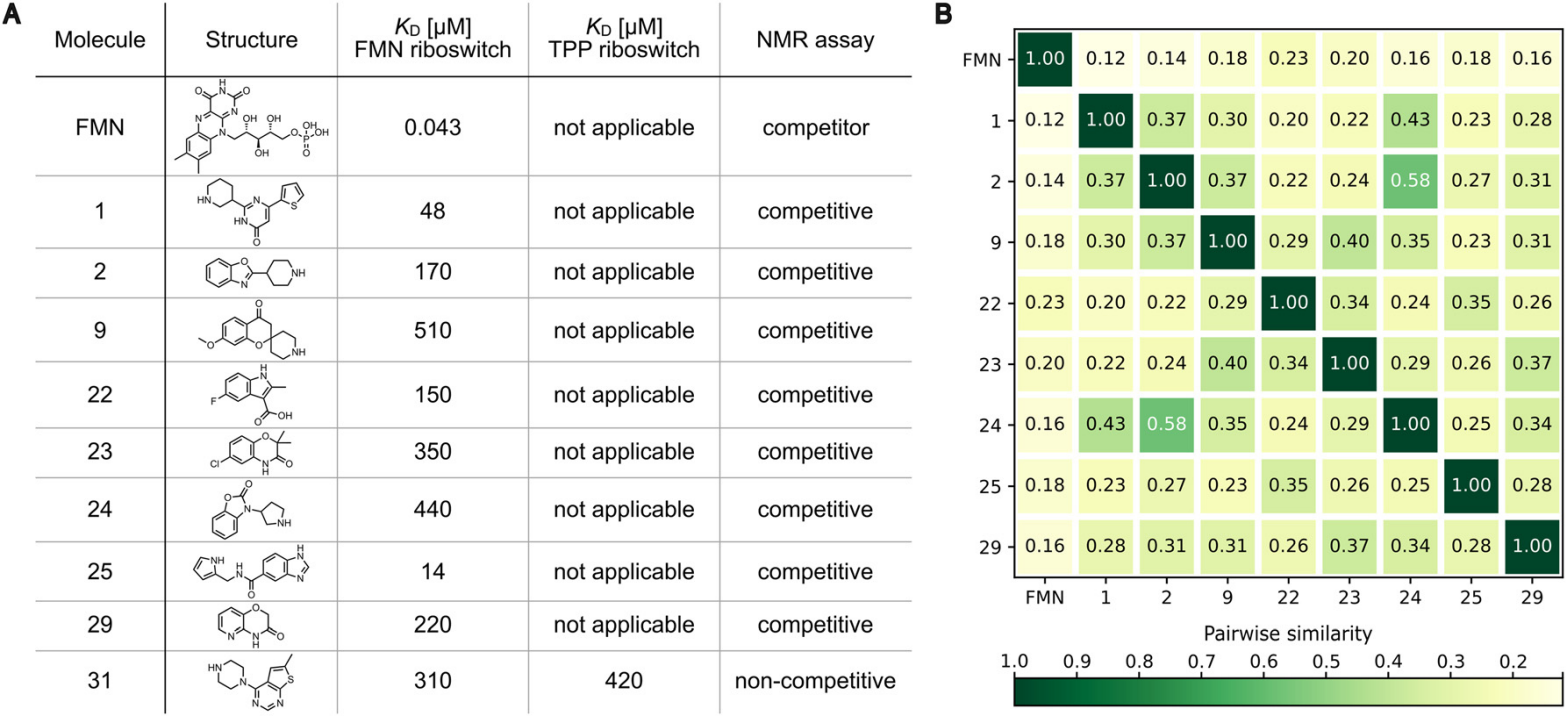
Overview of identified FMN riboswitch-binding fragments. A) Classification of fragments as competitive or non-competitive binders to the FMN riboswitch based on competitive NMR assays. Apparent binding affinities (steady-state fits) were determined by biolayer interferometry (BLI). B) Pairwise Tanimoto similarity for all fragments binding competitively to the FMN riboswitch as well as FMN and 1. Low Tanimoto coefficients (max. 0.58) across all pairwise comparisons excluding self-comparisons indicate a chemically diverse set of fragments with unique scaffolds.

To assess whether the identified fragments bind specifically to the riboswitch and to determine if they occupy the natural metabolite binding pocket, we established an NMR-based displacement assays employing the cognate ligands of the respective riboswitches as competitors. These assays, adapted from an RNA fragment screening approach previously described by Berg, H. *et al.*, provide a ternary readout of binding site specificity derived from the analysis of multiple NMR experiments.^45^ Given the substantial difference in binding affinities between the competitor ligands (nanomolar range) and the fragments (micromolar to sub-millimolar range), fragments occupying the metabolite binding pocket were expected to be displaced upon equimolar addition of the cognate ligand (FMN, SAM or TPP) and thus labelled as competitive ligands. Accordingly, fragments that retained binding signals in the presence of cognate ligands were classified as non-competitive binders (Fig. 5A). Additionally, the NMR experiments enabled the exclusion of non-binding fragments. For none of the 21 tested fragments aggregation was observed. Further, eight fragments showed binding to the FMN riboswitch. Among these, seven were displaced by FMN and were thus classified as competitive binders (Fig. 6A). The non-competitive fragment 31 demonstrated also non-competitive binding against the TPP and SAM-I riboswitches, indicating general non-specific RNA binding (Fig. S4).

Further, we performed a pairwise Tanimoto similarity analysis between the seven identified competitive FMN riboswitch-binding fragments as well as FMN and 1 using Morgan fingerprints. Low Tanimoto coefficients (ranging from 0.20–0.58 for the seven competitive fragments) indicate the discovery a of chemically diverse set of competitive binding fragments with unique scaffolds (Fig. 6B).

To evaluate whether the identified binders exhibit a bias toward specific charge states, we predicted the protonation states of all library compounds at pH 7.4 (the same pH as used in the BLI screening campaign) and analysed their charge distribution (Fig. S5). In the screening library (651 compounds), 54 compounds (8.3%) were predicted to carry a positive charge (+1: 53, +2: 1). This proportion was slightly higher in the primary BLI hits (4 of 35 compounds, 11.4%) but markedly enriched among the competitive ligands (3 of 7 compounds, 42.9%). This outcome is generally consistent with expectations, given the negatively charged phosphate backbone of RNA, however recent analysis of screening data has not indicated an overrepresentation of positively charged ligands among RNA binders.^18,46–48,53^ Although one might have expected to identify a larger number of negatively charged fragments due to the phosphate-binding site of the FMN riboswitch, this was not observed. Only a single negatively charged competitive binding fragment was identified. This might be because the design of effective phosphate bioisosteres remains challenging: they often suffer from poor stability, suboptimal physicochemical properties, or limited compatibility with fragment-like scaffolds.^49^

## Conclusions

Despite a growing interest in harnessing the pharmacological potential of mRNA elements, progress in developing reliable primary methods that can be broadly applied to diverse RNA targets for fragment-based lead discovery remains limited.^12,16,50^ While the potential of BLI for fragment screening has been demonstrated against protein targets and it has been used to measure RNA–ligand binding,^28,29^ it has yet to be employed in the identification of fragment binders for RNA targets.^26–29^

Riboswitch classes primarily identified in bacteria can regulate genes involved in essential cofactor biosynthesis, highlighting their potential as targets for the development of novel antibacterial agents. However, only limited efforts have been made to identify ligands containing scaffolds distinct from their natural ligands. In this study, we sought to evaluate BLI as a primary method to detect fragment binders of three riboswitches as a model system and thereby establish the feasibility of BLI in RNA-targeted fragment screening, potentially paving the way for its wider application in RNA-focused drug discovery.

Optimization of the assay conditions revealed that immobilization levels of the three riboswitches and relative response upon their interaction with small molecule binders depend on the Mg^2+^ concentration used for refolding and conducting the binding experiments (Fig. 2A and B). As the BLI response is proportionate to the biolayer thickness, these observations are broadly in agreement with the previous findings of a Mg^2+^ induced compact conformation of these riboswitches.^51,52^ In general, good agreement between the affinities derived from both steady state and kinetic fitting of these riboswitches against their weak and high-affinity binders with previous studies clearly demonstrates that BLI can be used for routine measurement of RNA–ligand binding. However, it is advisable to optimize assay conditions to obtain a good signal-to-noise ratio.

The BLI assays for the FMN and TPP riboswitch were stable enough over time to justify a fragment screening campaign. In contrast, the performance of the SAM-I riboswitch was found insufficient to be included in the fragment screening. The signal-to-noise ratio of the SAM-I riboswitch to 100 μM SAH was relatively low, with an average response of 0.02 nm at 1 h post-immobilization compared to 0.07 nm for the ribocil fragment 1 and 0.05 nm for thiamine. This is expected due to the relative low affinity of SAH (0.5 mM) compared to the other two control compounds (48 μM and 10 μM, respectively). Therefore, in order to use SAH as a control compound for the fragment screen, a higher concentration of SAH would have been needed to get a better signal. However, as we wanted to use the control compound in the same concentration as the fragments, this option was here not considered.

Compared to protein targets, the chemical space of RNA ligands is underexplored. Very few fragment screens against RNA targets have been reported to date.^16,18,21,23,47^ While some general properties of RNA ligands have been suggested,^46,51^ these are derived based on relatively small data-sets. To not introduce bias based on previous limited chemical space exploration, we therefore opted to screen a diverse fragment library.

The fragment screening campaign initially identified 35 primary hits, out of which 31 showed dose-dependent behaviour, mainly for the FMN riboswitch. Using a complementary NMR assay, out of a subset of 21 fragments, seven were shown to bind specifically and competitively to the FMN riboswitch while one (fragment 31) showed non-specific binding to all investigated riboswitches. This high dropout rate was largely caused by taking forward fragments with an atypical response at >50 μM (3, 4, 5, 11, 15, 16, 19, 20). It turned out that all of these were false positives when verified using NMR.

To the best of our knowledge, this is the first successful use of BLI for identifying competitive fragments against an RNA target. Indeed, we are also not aware that related methods like surface plasmon resonance (SPR), or grating-coupled interferometry (GCI) have been used for this purpose. Further, this is the first study reporting fragment binders against the FMN riboswitch. Moreover, the identified fragments have diverse scaffolds. Among these, fragment 25 has the highest affinity of 14 μM, whereas the affinities of the remaining fragments are in the sub-millimolar range. Thus, they are valuable starting points to develop potent ligands to further explore the FMN riboswitch as a target for antibiotics.

Interestingly, positively charged compounds were enriched among the identified competitive fragments (Fig. S5). This might be expected based on the negative nature of RNA, but was not observed in previous screens.^18,46–48,53^ This highlights that chemical space of RNA ligands is still underexplored and deriving general rules based on the limited available data might be premature.

In the case of the TPP riboswitch, despite more than 20 fragments eliciting a relative response of >0.05 nm, only one fragment gave a positive dose response and was confirmed as a non-competitive binder. However, others have previously identified fragment binders against the TPP riboswitch using a ligand displacement assay or a chemical probing method.^21,23^ Our library contained 6 fragments that were similar to five of the previously identified TPP riboswitch hits (Table S3). All of these previously identified hits were either non-specific or weak binders (high sub-millimolar range) or had poor solubility. In addition, despite having Tanimoto coefficients >0.7, the related library compounds except of AA3B10 are structurally sufficiently different to make binding to RNA unlikely. We therefore assume that the BLI screen rightly did not identify any of these analogues as hits. In case of AA3B10 the affinity might will be under the detection limit of the BLI assay. The absence of any competitive binder for the TPP riboswitch in this study depicts an RNA target dependent success of BLI in identifying fragment ligands. In a recent structure-based druggability analysis, we identified the FMN riboswitch binding site as being druggable while the druggability of the TPP riboswitch binding site was found to be conformation dependent. Based on these findings, we speculate that the expected hit rate for the TPP riboswitch is lower than for the FMN riboswitch and that the library we used here was not compatible with the TPP riboswitch binding site.

Altogether, this study for the first time demonstrates the use of an optical biosensor-based method as a primary method to identify fragment binders of RNA targets. Our results suggest that due to its easy setup, speed and throughput, BLI offers an attractive means for fragment screening campaigns against RNA targets, although target specific optimization is required. Generally, it should be possible to immobilize different RNA targets with a biotin tag on a streptavidin-based BLI sensor at moderate to high density. Since the tag is located at the end of the construct and very small, it is not expected to interfere with ligand binding outside this region. Our study shows that varying Mg^2+^ concentration could help to improve the immobilization levels, which in turn will enhance the overall performance of the assay for a given RNA target. However, it is advisable to evaluate the assay performance to decide if BLI is an appropriate primary method to identify RNA fragment binders. In our view, the major limitation of BLI in this regard is 1) to obtain a sufficiently high signal-to-noise ratio for binders with sub-millimolar affinity and 2) to discriminate true binders from non-binders in a dose response assay, due to atypical response at higher concentration (>50 μM). We find that the latter can be addressed by combining BLI with an orthogonal NMR assay.

## Experimental

### Compounds and reagents

All reagents employed in this study, including FMN, SAM, TPP, SAH and thiamine were purchased from Thermo Scientific™ unless specified otherwise. Fragment 1 was synthesized in-house (see SI for details). Dose–response verified fragments were purchased from the Otava chemicals or enamine. DNA sequences were purchased from GenScript. Super streptavidin biosensors (SSA) were purchased from Sartorius.

### DNA templates and RNA synthesis

DNA sequences were designed as per the previously published sequences of the SAM-I^41^ and TPP^23^ riboswitch or the FMN riboswitch (Table S2). The DNA templates required for *in vitro* transcription were prepared by annealing an equimolar mixture of the synthetic single-stranded DNA (bottom strand) with the T7 promoter sequence (Table S2) at 95 °C for 2 min followed by snap-cooling on ice for 5 min. For the SAM-I riboswitch, the transcription template was prepared by PCR using primers directed against the T7 promoter and the HδV ribozyme in the plasmid as described previously.^41^ The reaction mixture for *in vitro* transcription (IVT) contained 100 mM HEPES–NaOH (pH 8), 40 mM DTT, 2 mM spermidine, 50 mM MgCl_2_, 8 mM NTP-mix at pH 8, 10% DMSO, 100 nM annealed DNA template or about 40 μg mL^−1^ of PCR amplified dsDNA and 0.2 mg mL^−1^ of T7 RNA polymerase. The transcription reaction was incubated at 37 °C for at least 4 h. After removing magnesium pyrophosphate precipitates *via* centrifugation, the supernatant was filtered through 0.2 μm filter and the RNA was isolated using acidic ethanol precipitation. The RNA pellet was resuspended in 2× RNA loading dye (NEB) and was purified using 10% urea-PAGE. Bands containing the transcript of interest were visualized under UV shadow at 260 nm and excised. The RNA was eluted using three runs of passive diffusion at 4 °C, each involved incubation of crushed gel pieces in a freezer for 1 h, followed by elution in 0.3 M sodium acetate (pH 5.2) for at least 3 h at 4–8 °C. After each round of elution, the RNA concentration was estimated using a NanoDrop spectrophotometer and 10 μL aliquots were stored at −80 °C. Between 5–10 mg of purified RNA was obtained from a 12.5 mL reaction.

### RNA biotinylation

RNA was biotinylated using periodate oxidation of the 3′ end.^54^ Briefly, the reaction mixture containing 100 mM sodium acetate (pH 5.2), 5 μM RNA and 2.5 mM NaIO_4_ was incubated on ice for 50 min. The reaction was stopped by extracting RNA using ethanol under acidic conditions. The oxidized RNA was biotinylated by resuspending 1.5 nmol of RNA in 10 mM EZ-Link®Hydrazide-PEG-Biotin solution. After 2 h at 37 °C, NaBH_4_ was added to a final concentration of 33 mM at pH 8 and the reaction was further incubated on ice for 30 min. The reaction was terminated by extracting RNA using ethanol under acidic conditions and excess biotin was removed by washing twice with 70% (v/v) ethanol. Biotinylated RNA was aliquoted and stored at −80 °C.

### BLI sensor preparation and immobilization

BLI measurements were performed on an Octet RED96e (Sartorius) instrument. Biotinylated FMN and SAM-I riboswitch at 1 μM and TPP riboswitch at 250 nM were loaded on SSA biosensors previously equilibrated with the immobilization buffer (10 mM Tris, pH 8, 50 mM KCl, 150 mM NaCl) with varying concentrations of MgCl_2_ (0.2 mM, 0.5 mM, 1 mM, 2 mM, 5 mM and 10 mM) for 3 min. For screening and dose–response experiments, immobilization was carried out at 10 mM MgCl_2_ concentration. After immobilization, sensors were blocked with 10 μg mL^−1^ biocytin followed by washing with immobilization buffer containing 10 mM of MgCl_2_. For screening and dose–response experiments, typically, we obtained immobilization levels between 2.5–3 nm.

### Characterization of riboswitch–ligand binding using BLI

For initial optimization of BLI assay conditions, immobilized riboswitches were tested for response against known weak ligands (fragment 1, SAH, and thiamine) at 100 μM and high-affinity ligands (FMN, SAM, TPP) at 5 μM in the reaction buffer (0.01 M HEPES pH 7.4, 0.15 M NaCl, 0.005% v/v Surfactant P20) containing 2 mM MgCl_2_ unless specified otherwise. The affinity of these ligands was estimated by applying steady state and kinetic fit models on serial dilution series. The assay settings were as follows: baseline measurement 60 s; association time 120 s and dissociation time 180 s. The resulting data were processed using the reference subtracted method of the Octet system data analysis software.

### Z′-Factor calculation

The *Z*′-factor is a commonly used statistical parameter for assessing the robustness and quality of the underlying assay.^44,55^ It incorporates the signal dynamic range and the data variation of the positive and negative controls. Assays providing *Z*′-factors > 0.5 are considered robust and reliable. *Z*′-Factors between 0.5 and 0 are considered marginal and may require some optimisation. *Z*′-Factors below 0 are not acceptable.

The *Z*′-factors were determined using the weak binders as positive controls, namely 1 for the FMN riboswitch, SAH for SAM-I riboswitch, and thiamine for the TPP riboswitch and buffer only wells as negative controls. Further, 10 replicates over time were used in the calculations according to the following equation:
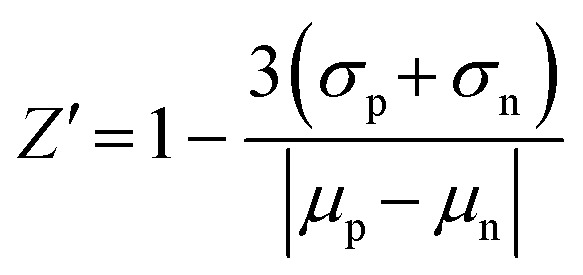
where *μ*_p_ is the mean of the positive control, *μ*_n_ is the mean of the negative control, *σ*_p_ is the standard deviation of the positive control, and *σ*_n_ is the standard deviation of the negative control.

### Fragment screening library

For fragment screening, the University of Bergen Biophysics, Structural Biology and Screening facility (BiSS) fragment library containing 651 fragments was used.^26^ Briefly, the library is a subset of the OTAVA solubility fragment library which was filtered to exclude similar compounds and compounds with unwanted functionalities. The fragments have a mean molecular weight of 202 Da, an average clog *P* (calculated partition coefficient) of 1.52 and contain on average two ring systems, one hydrogen-bond donor, two hydrogen-bond acceptors, and two rotatable bonds.

### General BLI screening setup

Screening plates were prepared by adding 10 μL of a 2 mM DMSO stock solution of library compound to 190 μL of 1.05× HBS-P+ buffer, pH 7.4 (Cytiva, BR100671) supplemented with 2.1 mM MgCl_2_ in 96 well plates (Greiner), leading to a final DMSO concentration of 5% (v/v) and a fragment concentration of 100 μM. Column 1 and 12 contained a buffer with 2% DMSO and were used for equilibrating the sensors and dissociation, respectively. The assay settings were as follows: baseline measurement 60 s; association time 60 s and dissociation time 100 s. As a result, we were able to screen each plate against the reference ligand (dsDNA) and two RNA targets (FMN and TPP riboswitch) in 2 h. The resulting data were processed using the reference-subtracted method of the Octet analysis software. To monitor the structural integrity of the riboswitches, we measured the cognate binders and no-ligand controls at the end of each screening plate. We found that the typical ligand response did not fall below 0.05 nm, suggesting that screening was conducted under good assay conditions.

### Dose–response assay

Selected fragments from the initial screening were evaluated in dose–response titrations using eight serial concentration series ranging from 200–1.56 μM using the same conditions as in the screening setup. The reference subtracted data were further analysed in the Octet system data analysis software to estimate the affinity using either a steady state model or applying a global fit to 1 : 1 kinetic model. Briefly, the 1 : 1 kinetic model uses the following mathematical description of the interaction between the ligand and the analyte:
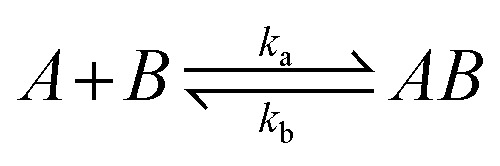

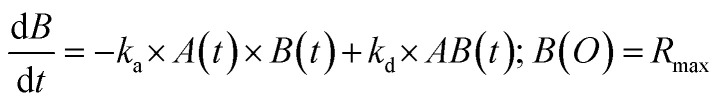

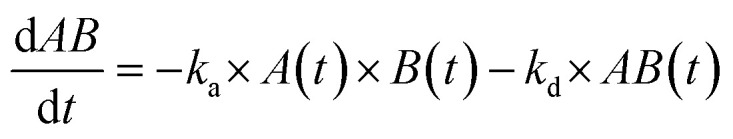
where *A* is analyte concentration, *B* is amount of unbound ligand binding sites, *AB* is the amount of complex formed, *k*_a_ is the association rate constant (M^−1^ s^−1^), *k*_d_ is the dissociation rate constant (s^−1^), *t* is the time (s), and *R*_max_ is analyte binding signal (nm) when all available ligand sites are occupied (at >10*K*_D_). The kinetics derived affinity (*K*_D,kin_) was calculated as the ratio of *k*_d_ to *k*_a_ using global fitting. The steady state affinity (*K*_D,stead_) represents the system at the equilibrium and was derived from the plot of response *versus* increasing concentration of the analyte. For the fitting, data points with responses lower than what was observed in the absence of ligand were rejected.

### NMR assay

All NMR spectra were recorded at 298 K using a Bruker AVANCE NEO 600 MHz spectrometer (Bruker BioSpin, Zürich, Switzerland) equipped with a SampleJet and a QCI-P CryoProbe, with samples prepared in 3 mm NMR tubes. Untagged RNA prepared *via* IVT was buffer exchanged into KPi buffer (25 mM KPi, 50 mM KCl, 5 mM MgCl_2_, pH 6.2) using a 3k MWCO filter (Merck), refolded by heating at 95 °C for 2 min, and cooled on ice for 30 min. The RNA concentration was determined using a NanoDrop spectrophotometer and adjusted to 29.4 μM with KPi buffer.

Samples were prepared as singletons. Ligands were dissolved in DMSO-*d*_6_ at 10 mM. Ligand-only samples were prepared by mixing ligand solution with D_2_O and KPi buffer to obtain a sample with 500 μM ligand concentration in 5% DMSO-*d*_6_ and 10% D_2_O. Ligand–RNA samples were prepared at a 1 : 20 RNA-to-ligand ratio and samples indicating binding behaviour were further probed for competitive binding by supplementing the sample with 80 nmol competitor (40 mM FMN, TPP or SAM in 5% DMSO-*d*_6_, 10% D_2_O, and 85% KPi buffer) and re-analysed. The Mg^2+^ concentration in all experiments was 4.25 mM. The spectra were superimposed, aligned, and normalized using the residual 1H resonance from the DMSO-*d*_6_ solvent signal as reference.

Fragment binding was evaluated using a combination of NMR techniques: waterLOGSY (pulse sequence ephogsygpno.2, NS = 256), *T*_2_ relaxation decay (cpmg_esgp2d, NS = 64, mixing time 5 ms and 200 ms), and chemical shift perturbation (CSP) analysis (noesygppr1d, NS = 32). Fragments were classified as binders if they exhibited a LOGSY factor greater than 1 and met at least one of the following criteria: a ≥60% reduction in *T*_2_ relaxation decay or a CSP exceeding 6 Hz.^46^

Ligands were considered competitive if upon addition of a known competitor, they showed a LOGSY factor below 1 and at least one of the following indicative changes: a reduction in *T*_2_ relaxation decay of less than 60% or a CSP below 6 Hz.

### Cheminformatics analysis

Pairwise molecular similarity analysis was performed using in house Python scripts based on RDKit (version 2025.03.6). Molecules were represented by Morgan fingerprints with a radius of 2 Å. Pairwise Tanimoto similarity coefficients were then calculated between all compounds in the dataset based on these fingerprints, yielding a similarity matrix which was visualised with Matplotlib.

Likewise, the similarity search for compounds in the fragment screening library related to known TPP riboswitch fragments was carried out with RDKit using the same fingerprints as above.

The charge of the fragments at pH 7.4 was predicted using EPIK in Maestro (Schrodinger, version 13.9.138).

## Author contributions

Conceptualization: V. N. P., J. H., B. E. H., and R. B.; BLI experiments: V. N. P. and K. G.; NMR experiments: J. H.; compound synthesis: M. Z. and B. E. H.; formal analysis: V. N. P. and J. H., funding acquisition: R. B., B. E. H. and V. N. P.; project administration: R. B. and V. N. P.; supervision: R. B., B. E. H. and V. N. P.; visualization: V. N. P. and J. H.; writing – original draft preparation: V. N. P. and J. H.; writing – review and editing: V. N. P., J. H., M. Z., B. E. H. and R. B.; all authors have read and agreed to the published version of the manuscript.

## Conflicts of interest

There are no conflicts to declare.

## Supplementary Material

MD-OLF-D5MD00673B-s001

MD-OLF-D5MD00673B-s002

MD-OLF-D5MD00673B-s003

## Data Availability

Supplementary information: SI is provided in PDF format and includes: i) the BLI sensorgrams, ii) additional figures and tables with analysis of the library compounds, iii) ^1^H NMR spectra of the eight binding fragments shown in Fig. 6A, iv) synthesis procedures, NMR spectra and purity data for fragment 1. All NMR spectra for binding analysis are provided as .jdx files and the analysis as .xlsx file. See DOI: https://doi.org/10.1039/D5MD00673B. All additional data are available from the corresponding authors upon request.
