# Supplementary material for: Discovery of RNA-binding fragments using biolayer interferometry
Source: RSC Med Chem. 2025 Sep 19;16(11):5629–40. doi: 10.1039/d5md00673b (PMC12483148; doi:10.1039/d5md00673b)
Supplement: MD-016-D5MD00673B-s003 [file MD-016-D5MD00673B-s003.pdf]

Supplementary Information (SI) for RSC Medicinal Chemistry.
This journal is © The Royal Society of Chemistry 2025

Electronic Supporting information

Discovery of RNA binding fragments using Biolayer Interferometry

Vipul Navinchandra Panchal[a,*,$], Jan-Åke Husmann[a,$], Kaja Günther[a], Muhammad Zeeshan[b], Bengt Erik Haug[b], Ruth Brenk[a,c,*]

a) *Department of Biomedicine, University of Bergen, Jonas Lies vei 91, 5020 Bergen, Norway*
b) *Department of Chemistry and Centre for Pharmacy, University of Bergen, Allégaten 41, 5007 Bergen, Norway*
c) *Computational Biology Unit, University of Bergen, Thormøhlensgate 55, 5008 Bergen, Norway*

** Corresponding authors*
$ both authors contributed equally to this study

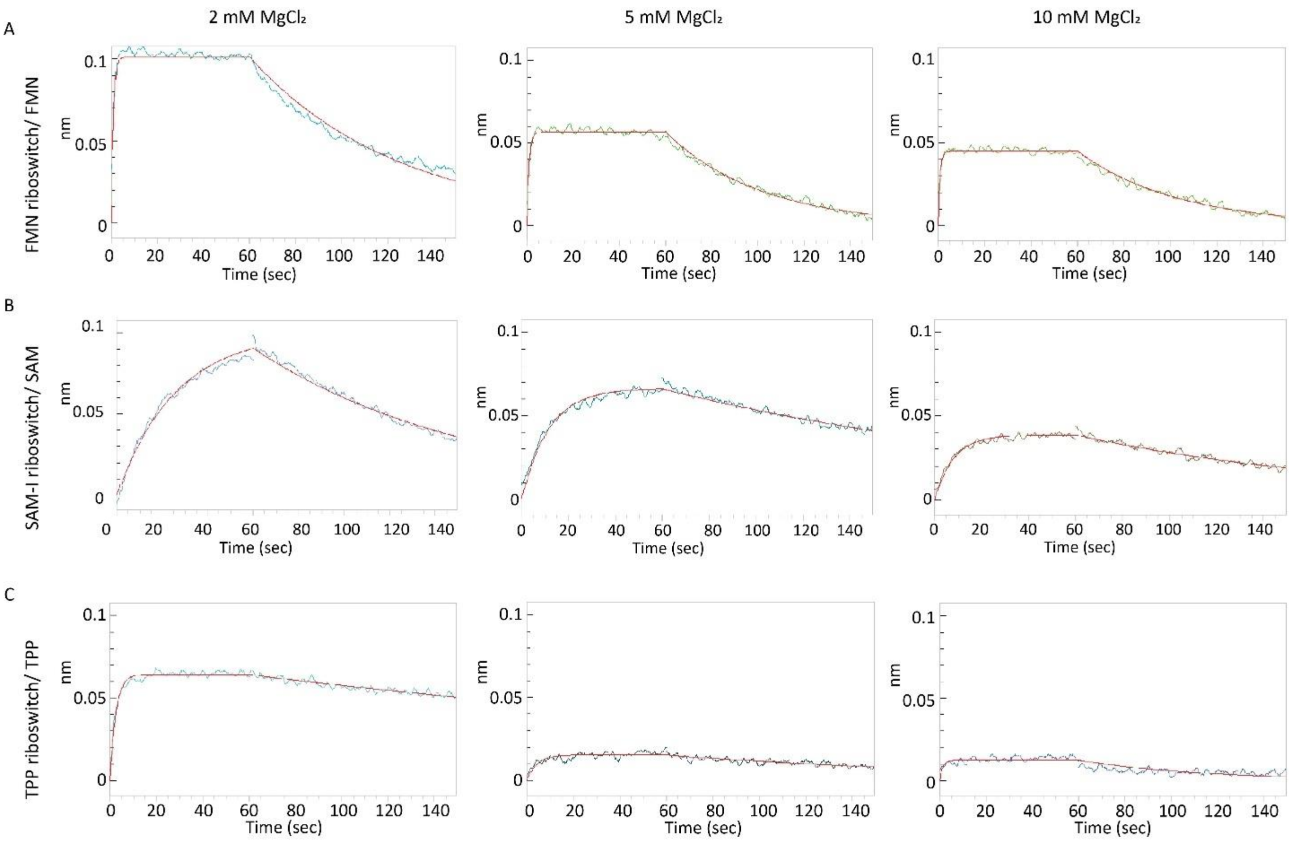

Fig. S1 Magnesium dependent response of the ligands to the investigated riboswitches. Sensograms of the A) FMN riboswitch-FMN, B) SAM-I riboswitch-SAM and C) TPP riboswitch-TPP pairs in the presence of 2 mM, 5 mM or 10 mM $MgCl_2$ (left to right) are shown.

Fragment	Kinetic fit (1:1 model)	Steady state fit (1:1)

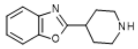

2

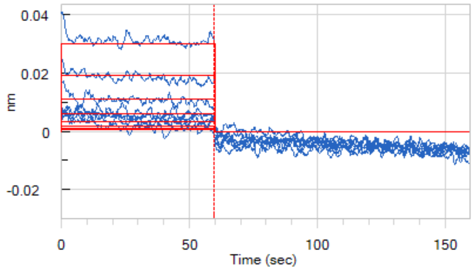

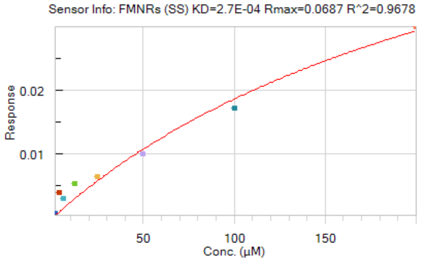

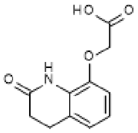

3

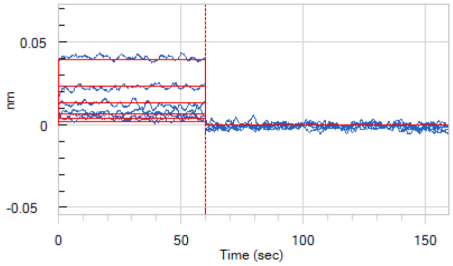

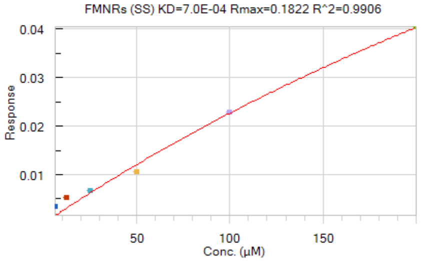

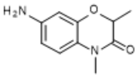

4

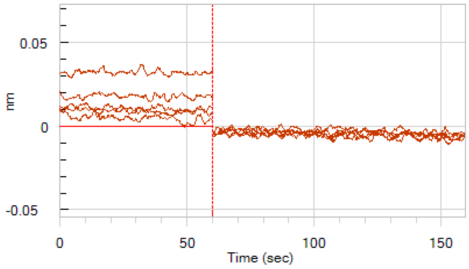

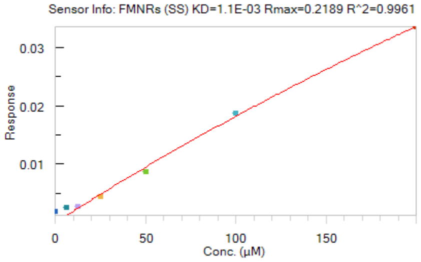

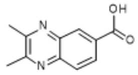

5

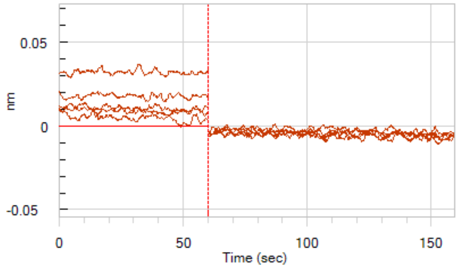

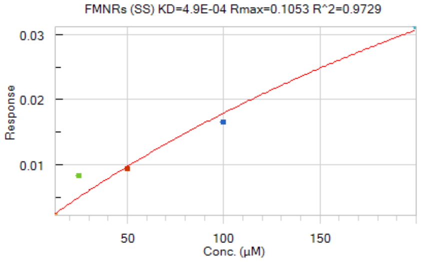

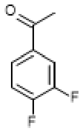

6

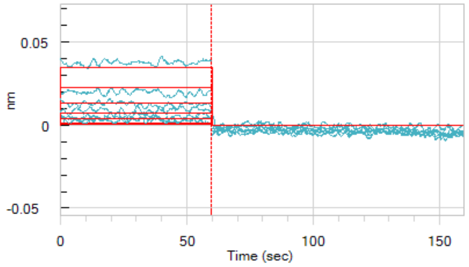

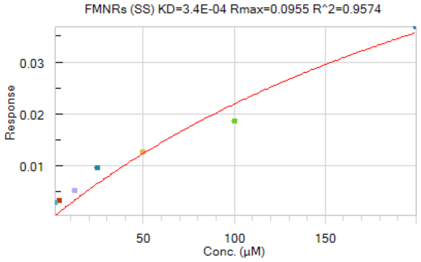

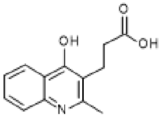

7

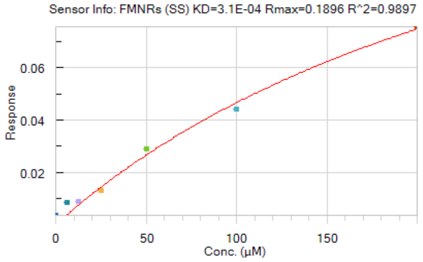

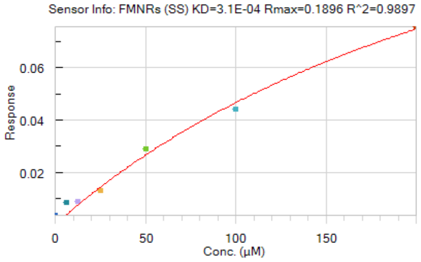

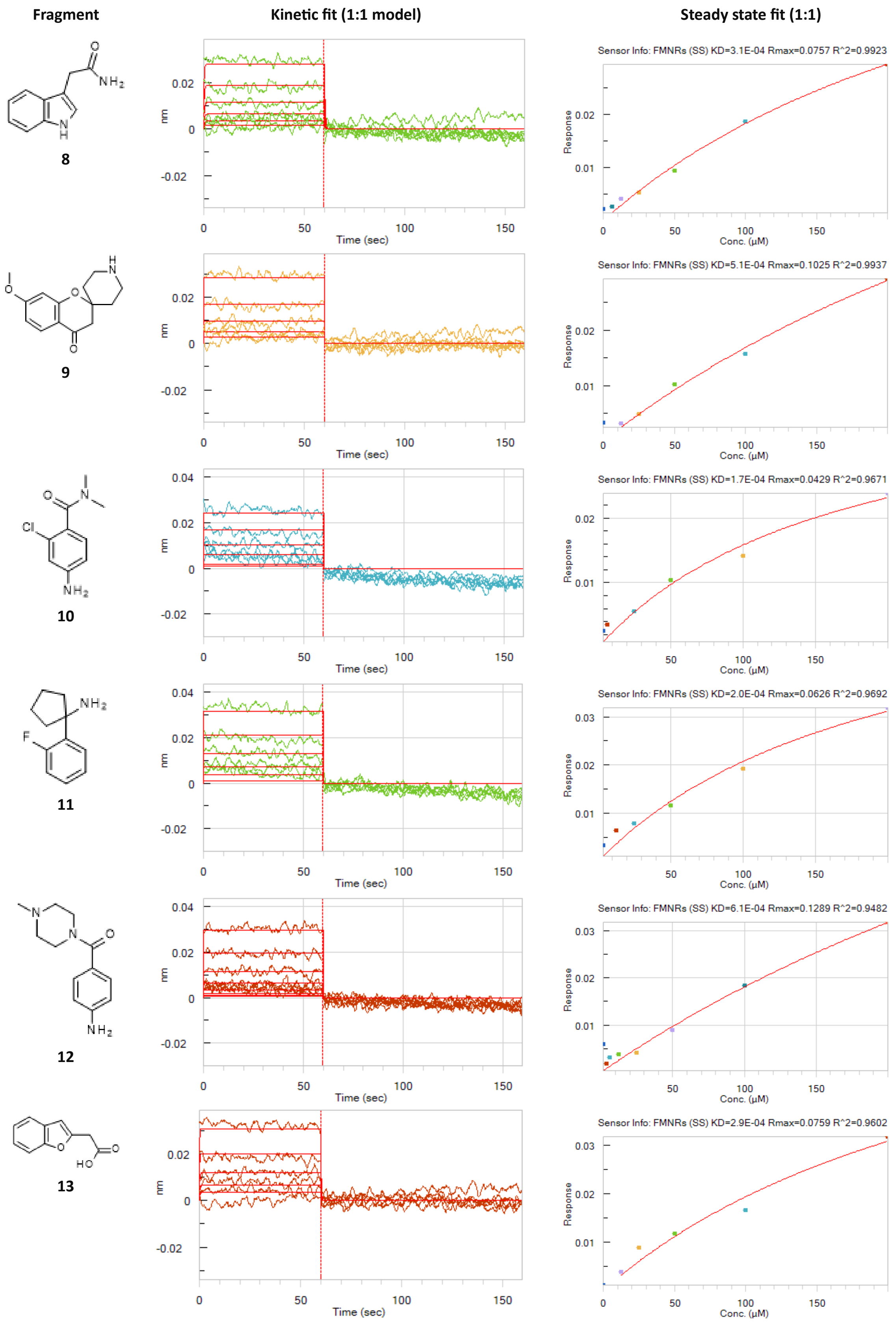
Fragment
Kinetic fit (1:1 model)
Steady state fit (1:1)
8
Sensor Info: FMNRs (SS) KD=3.1E-04 Rmax=0.0757 R^2=0.9923
9
Sensor Info: FMNRs (SS) KD=5.1E-04 Rmax=0.1025 R^2=0.9937
10
Sensor Info: FMNRs (SS) KD=1.7E-04 Rmax=0.0429 R^2=0.9671
11
Sensor Info: FMNRs (SS) KD=2.0E-04 Rmax=0.0626 R^2=0.9692
12
Sensor Info: FMNRs (SS) KD=6.1E-04 Rmax=0.1289 R^2=0.9482
13
Sensor Info: FMNRs (SS) KD=2.9E-04 Rmax=0.0759 R^2=0.9602
nm
Time (sec)
Response
Conc. (µM)

**Fragment**	**Kinetic fit (1:1 model)**	**Steady state fit (1:1)**
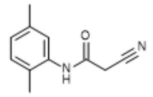 **14**	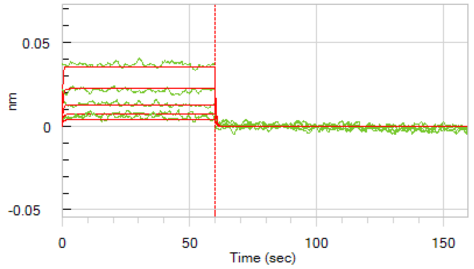	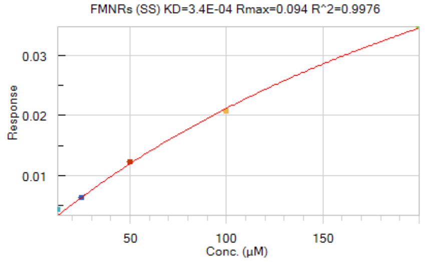
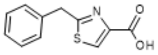 **15**	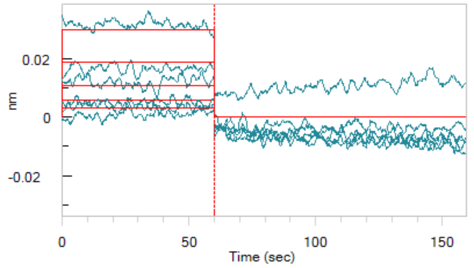	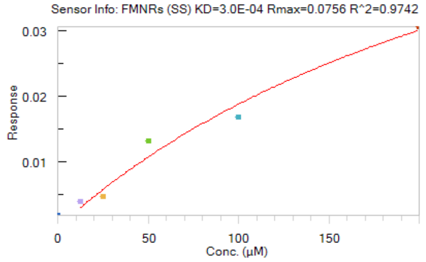
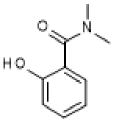 **16**	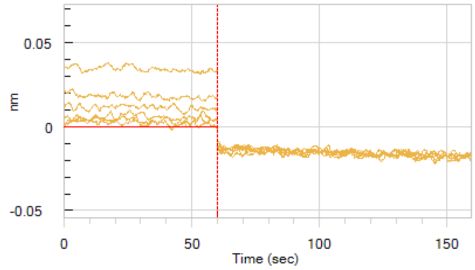	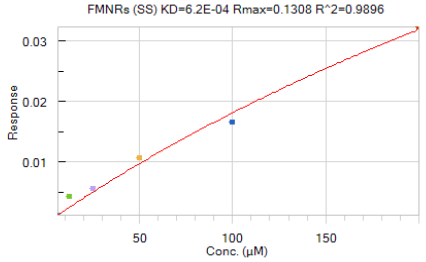
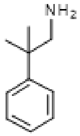 **17**	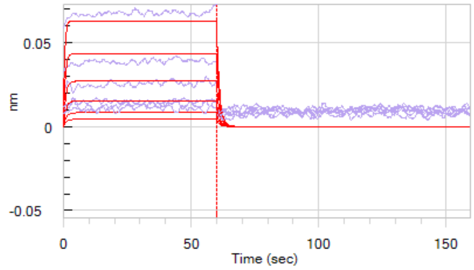	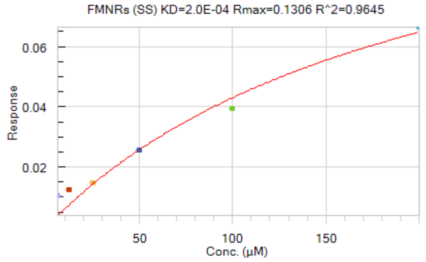
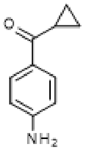 **18**	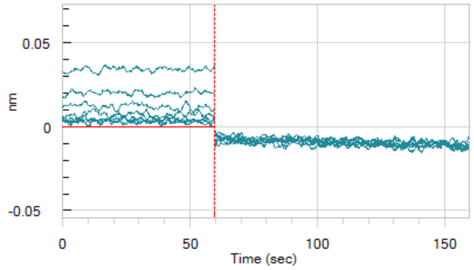	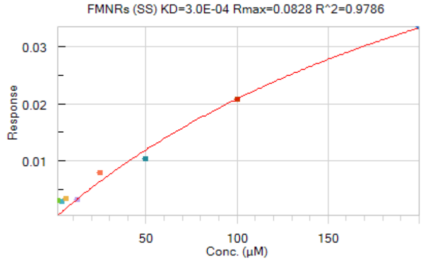
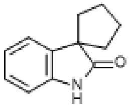 **19**	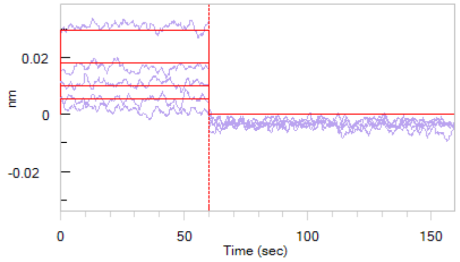	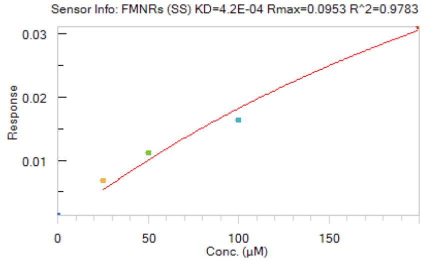

Fragment | **Kinetic fit (1:1 model)** | **Steady state fit (1:1)**

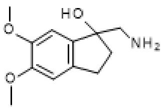

20

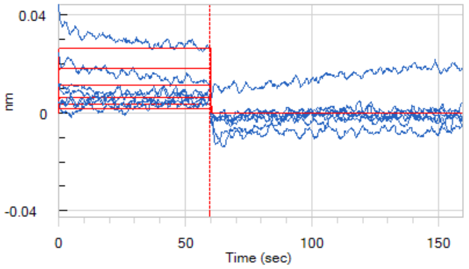

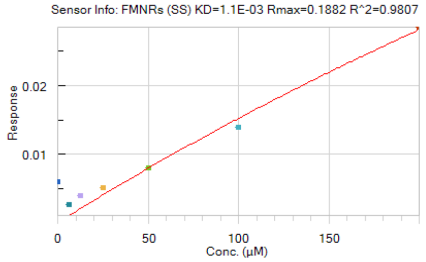

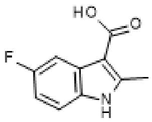

22

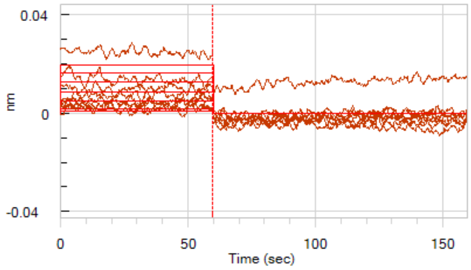

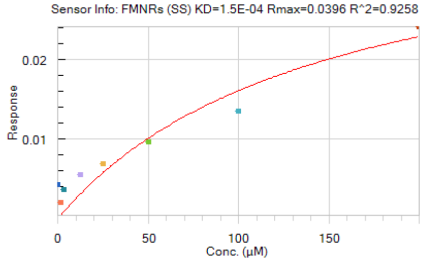

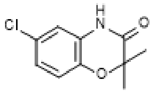

23

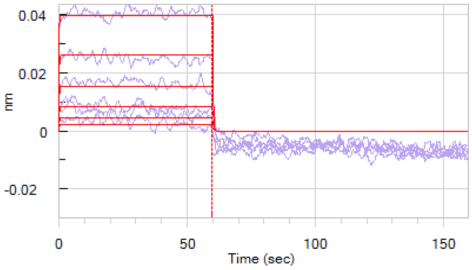

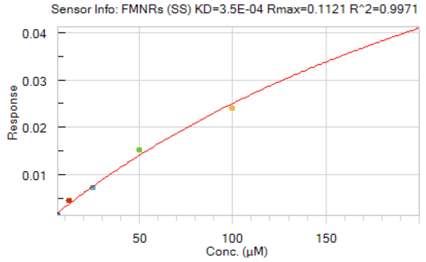

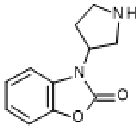

24

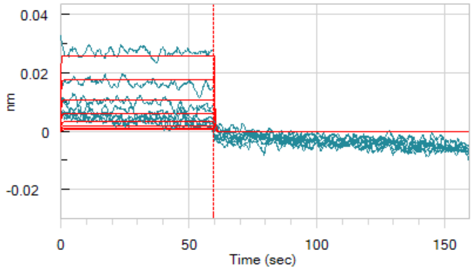

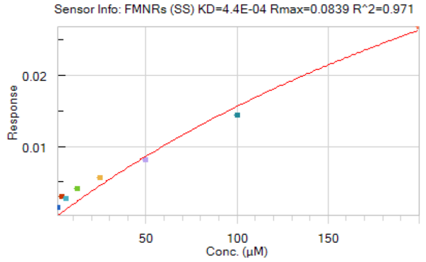

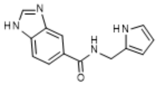

25

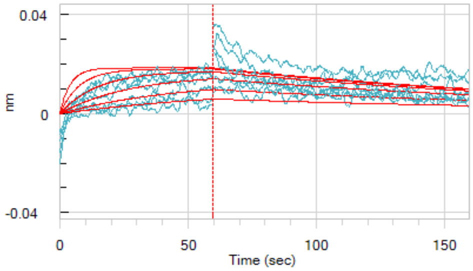

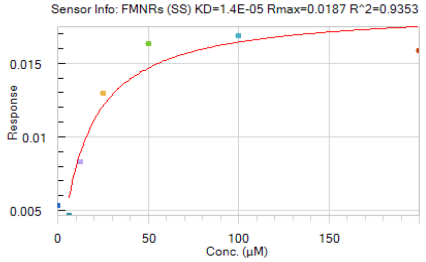

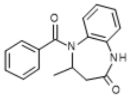

26

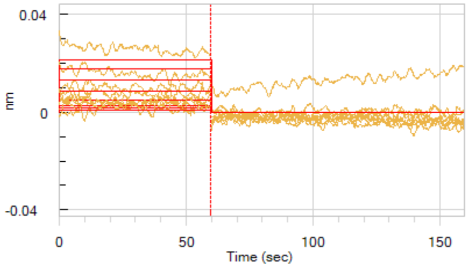

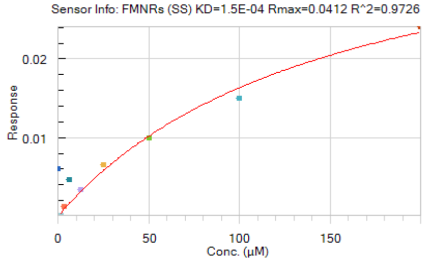

Fragment	Kinetic fit (1:1 model)	Steady state fit (1:1)

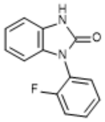

28

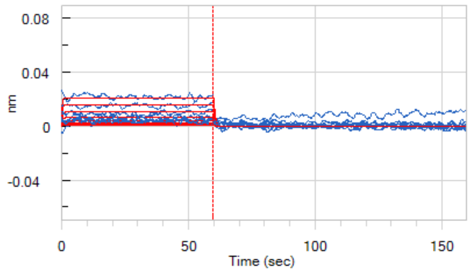

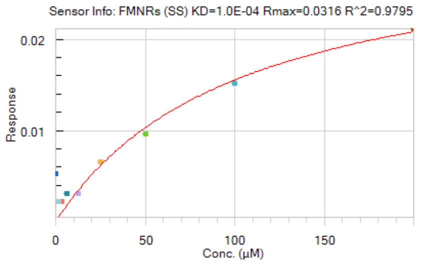

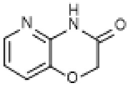

29

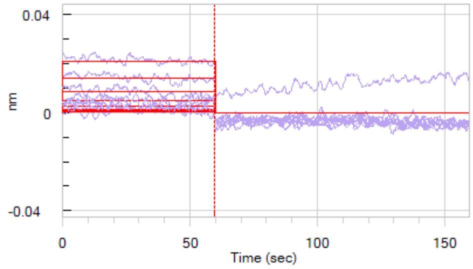

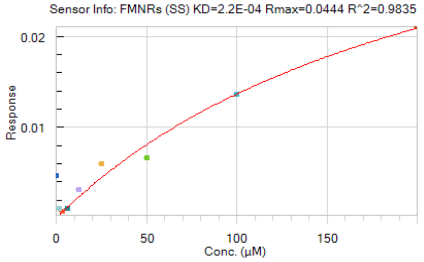

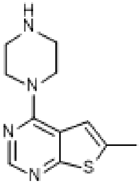

31

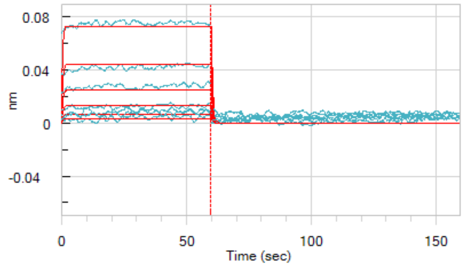

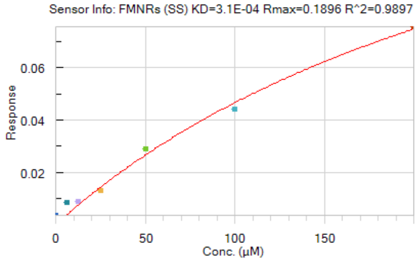

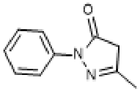

33

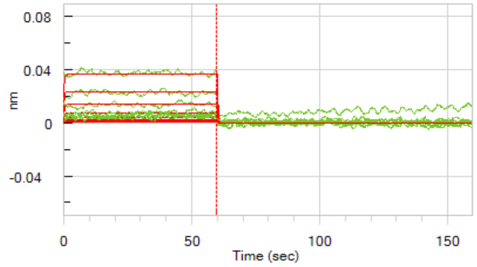

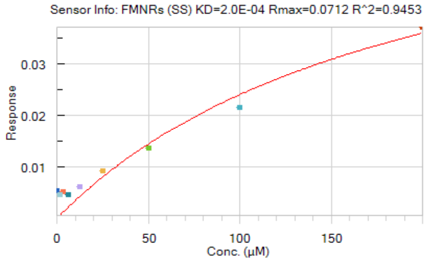

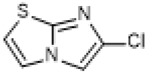

35

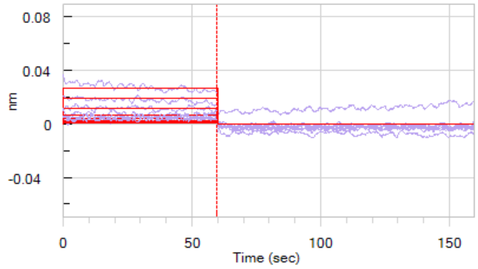

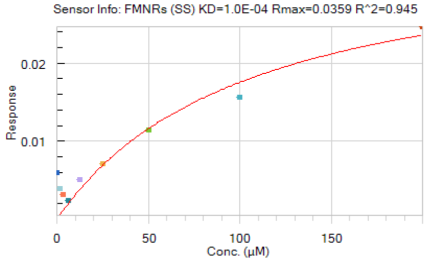

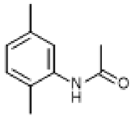

36

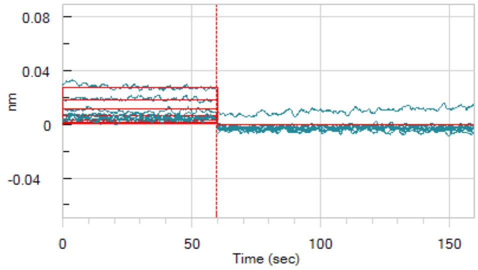

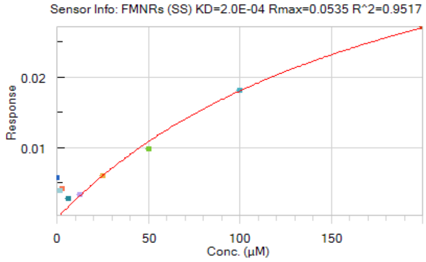

Fig. S2 Dose-dependent response of selected hits against the FMN riboswitch. Only compounds exhibiting a dose-dependent response with a goodness-of-fit (R^2) value of > 0.9 are shown. The chemical structures of the compounds are on the left, the 1:1 kinetic fits to the dose response curves against the FMN riboswitch are in the center, and the steady state binding analyses including the binding affinity (K_D), calculated R_{max}, and R^2 are presented on the right.

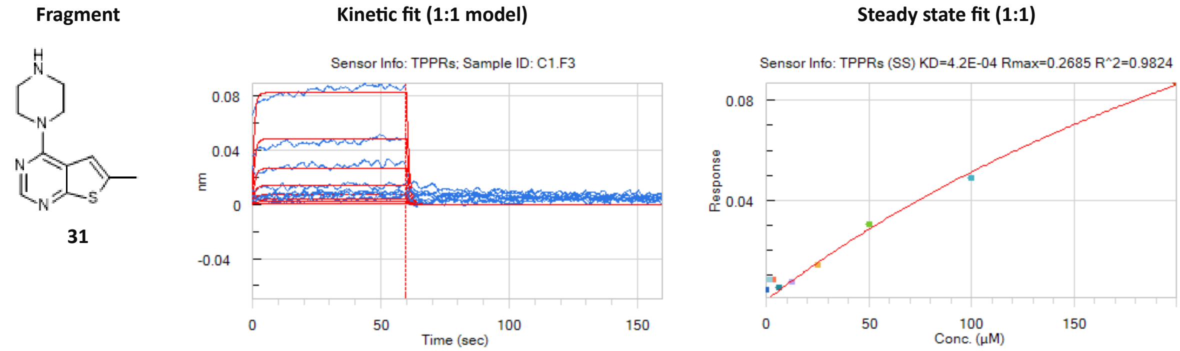

Fig. S3 Dose-dependent response of hit **31** against the TPP riboswitch. The chemical structure of **31** is on the left, a 1:1 kinetic fit to the dose response curves against the TPP riboswitch is in the center, and a steady state binding analyses including the binding affinity (K_D), calculated R_{max}, and R^2 is presented on the right.

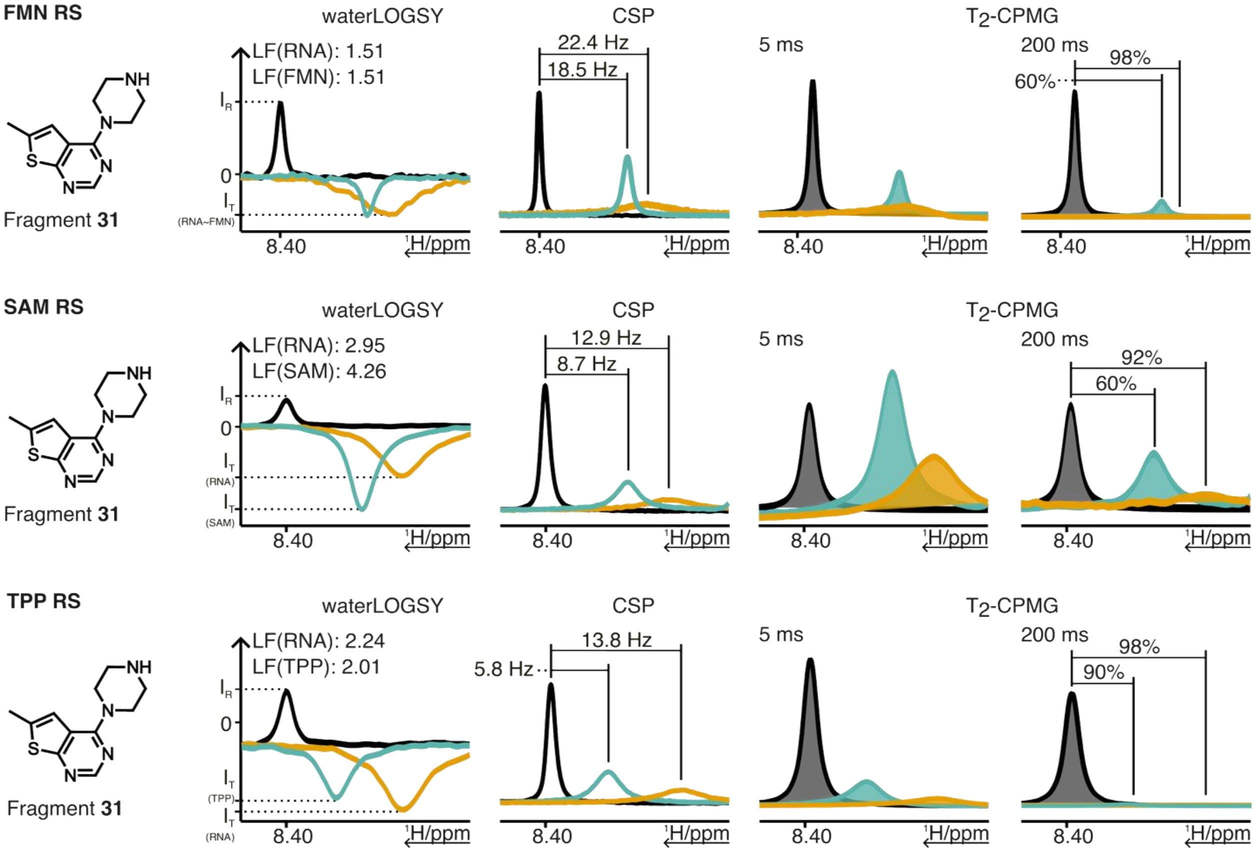

Fig. S4 Fragment **31** is a non-specific RNA binder. Fragment **31** shows non-competitive binding behaviour against the FMN, SAM-1 and TPP riboswitch using the developed competitive NMR assay. Colour coding: Fragment only (black), fragment in presence of RNA (orange), and fragment in presence of RNA and competitor FMN, TPP, or SAM (cyan). In all of the investigated riboswitches the high affinity ligands (FMN, SAM or TPP) were unable to outcompete **31**, and the NMR metrics stayed above the threshold (LOGSY factor > 1 plus at least one of the following criteria: a ≥ 60% reduction in T_2 relaxation decay or a CSP > 6 Hz) indicating continued binding of **31** towards the analysed RNA.

Charge	Screening library (651)	Primary BLI hits (35)	Competitive ligands (7)
**positive**	54 (8.3 %)	4 (11.4 %)	3 (42.9 %)
**neutral**	357 (54.8 %)	18 (51.4 %)	3 (42.9 %)
**negative**	171 (26.3 %)	54 (20.0 %)	1 (14.3 %)
**zwitterionic**	69 (10.6 %)	6 (17.1 %)	0 (0 %)

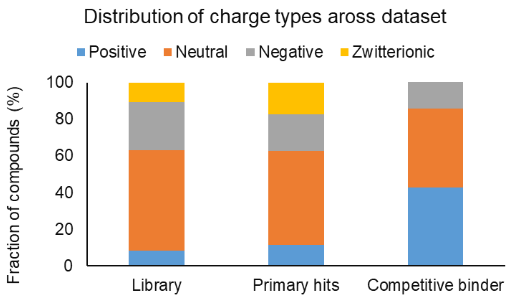

Fig. S5 Change in charge distribution from the initial screening library to the final identified competitive FMN riboswitch-binding fragments reveals a bias towards positively charge. While in the initial screening library only 8.3 % of the fragments are positively charged, 42.9 % of the competitive ligands are positively charged compounds.

A)

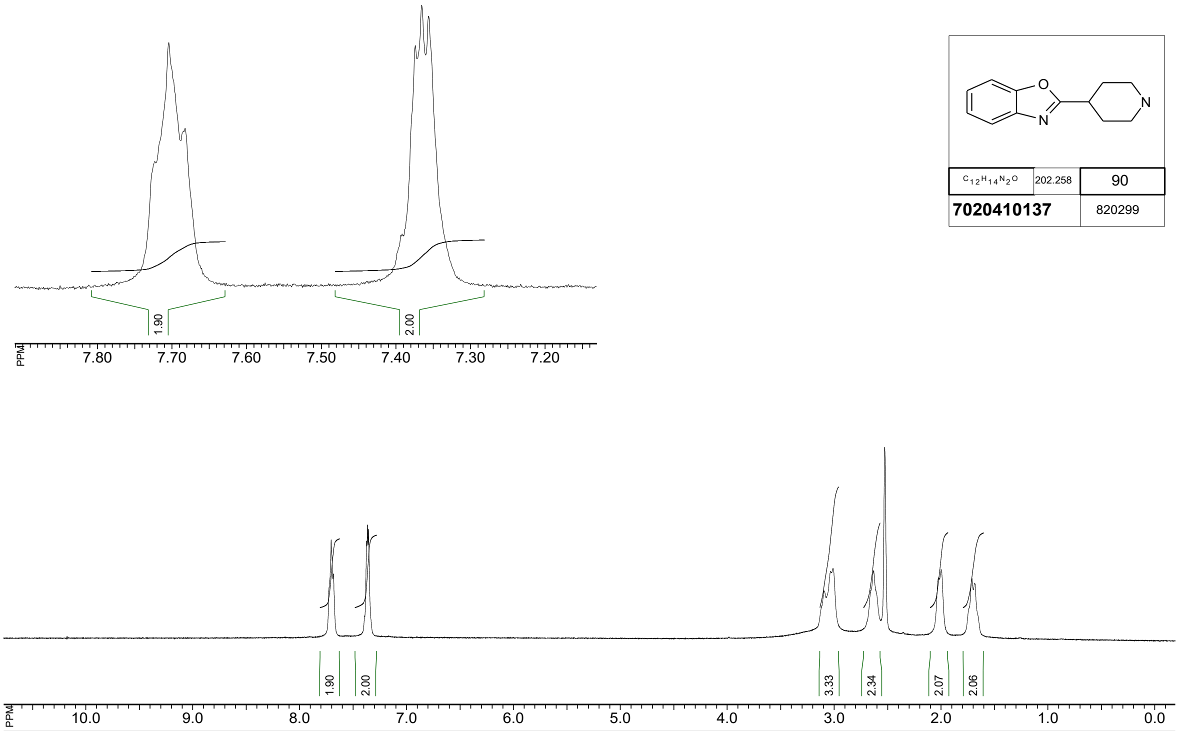

File name: 820299.fid	Operator: B	SF: 400.4493 MHz	NSC: 0	PW: 7.00 usec, RG: 18	SI: 32768
Date: 24-Feb-2014	Solvent: DMSO-d6	SW: 8000 Hz	TE: 294 K	AQ: 1.50 sec, RD: 0.00 sec	

B)

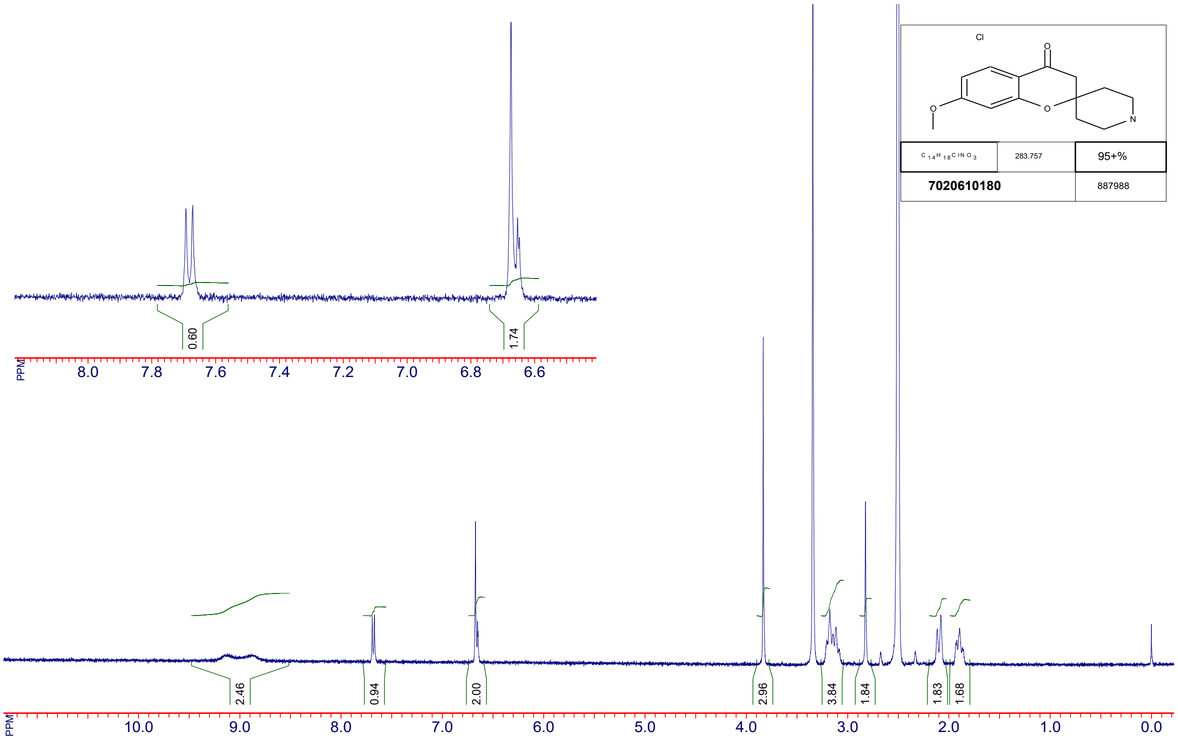

File name: yav3677.fid	Operator:	SF: 400.4494 MHz	NSC: 0	PW: 9.00 usec, RG: 22	SI: 32768
Date: 27-May-2009	Solvent: dmso	SW: 8000 Hz	TE: 293 K	AQ: 1.50 sec, RD: 0.00 sec	

C)

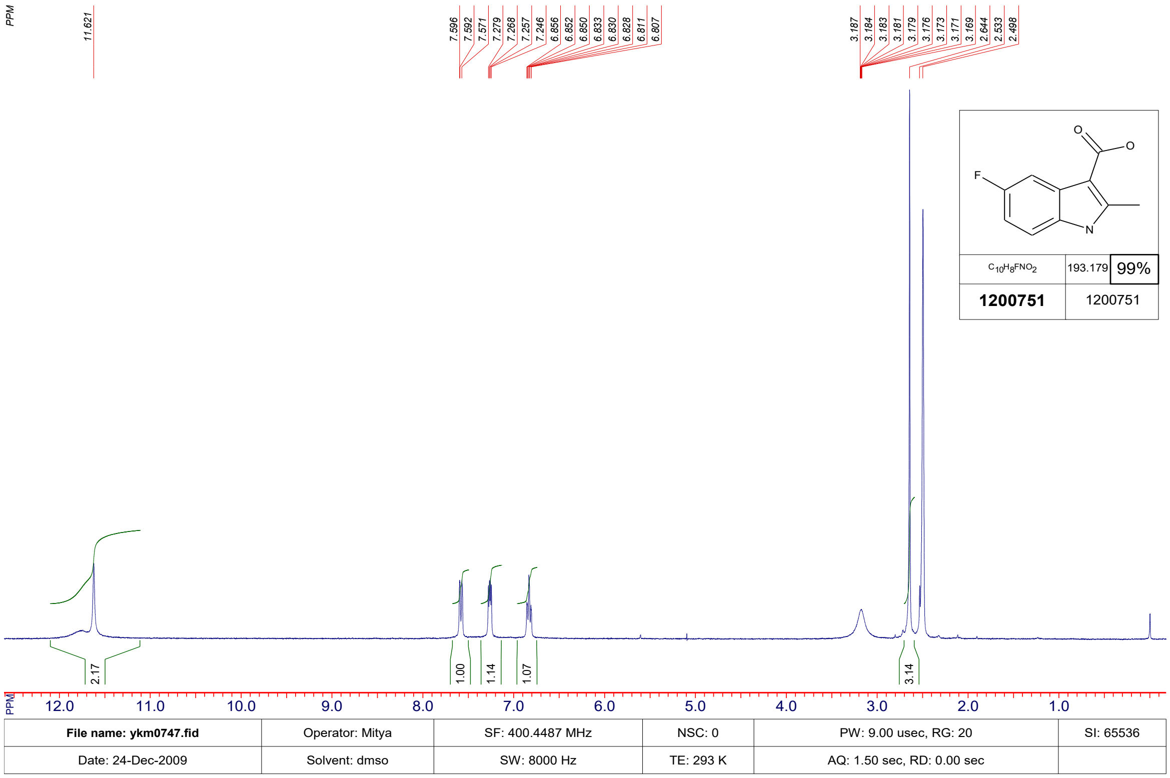
PPM
11.621
7.596 7.592 7.571 7.279 7.268 7.257 7.246 6.856 6.852 6.850 6.833 6.830 6.828 6.811 6.807
3.187 3.184 3.183 3.181 3.179 3.176 3.173 3.171 3.169 2.644 2.533 2.498
F
O
O
N
C10H8FNO2
193.179
99%
1200751
1200751
2.17
1.00
1.14
1.07
3.14
PPM 12.0 11.0 10.0 9.0 8.0 7.0 6.0 5.0 4.0 3.0 2.0 1.0
File name: ykm0747.fid
Operator: Mitya
SF: 400.4487 MHz
NSC: 0
PW: 9.00 usec, RG: 20
SI: 65536
Date: 24-Dec-2009
Solvent: dmso
SW: 8000 Hz
TE: 293 K
AQ: 1.50 sec, RD: 0.00 sec

D)

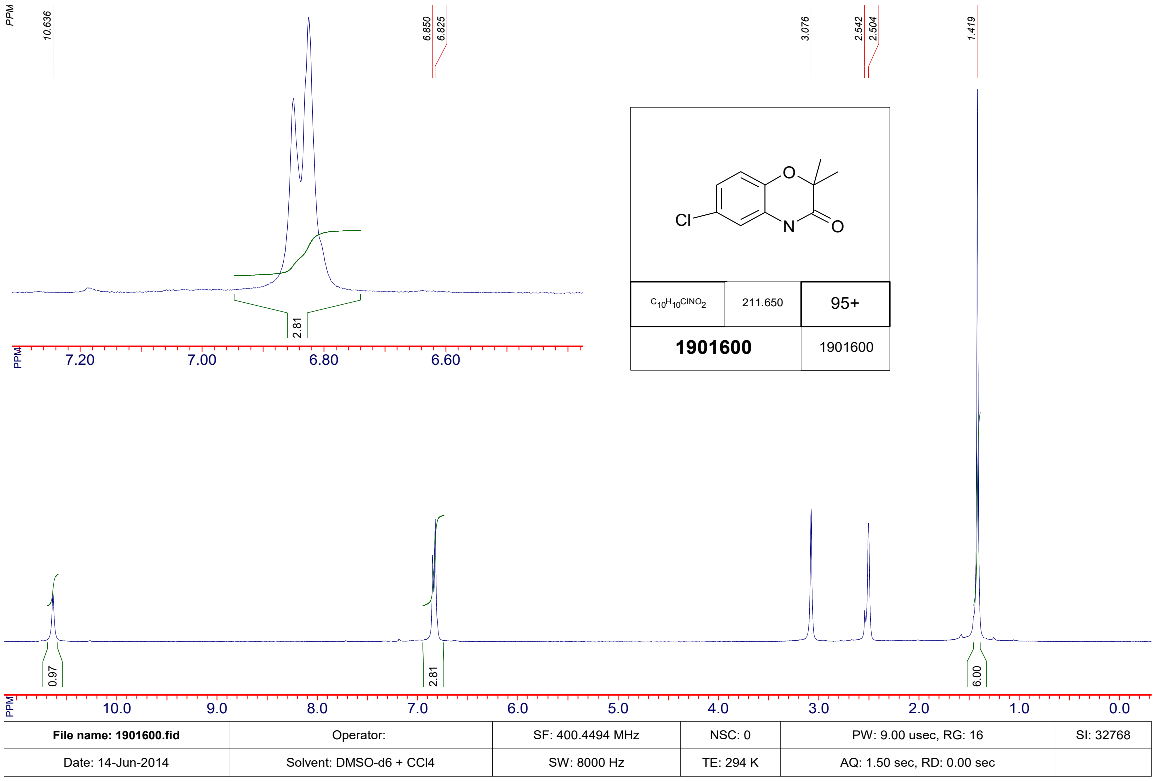
PPM
10.636
6.850 6.825
3.076
2.542 2.504
1.419
2.81
PPM 7.20 7.00 6.80 6.60
Cl
O
N
O
C10H10ClNO2
211.650
95+
1901600
1901600
0.97
2.81
6.00
PPM 10.0 9.0 8.0 7.0 6.0 5.0 4.0 3.0 2.0 1.0 0.0
File name: 1901600.fid
Operator:
SF: 400.4494 MHz
NSC: 0
PW: 9.00 usec, RG: 16
SI: 32768
Date: 14-Jun-2014
Solvent: DMSO-d6 + CCl4
SW: 8000 Hz
TE: 294 K
AQ: 1.50 sec, RD: 0.00 sec

E)

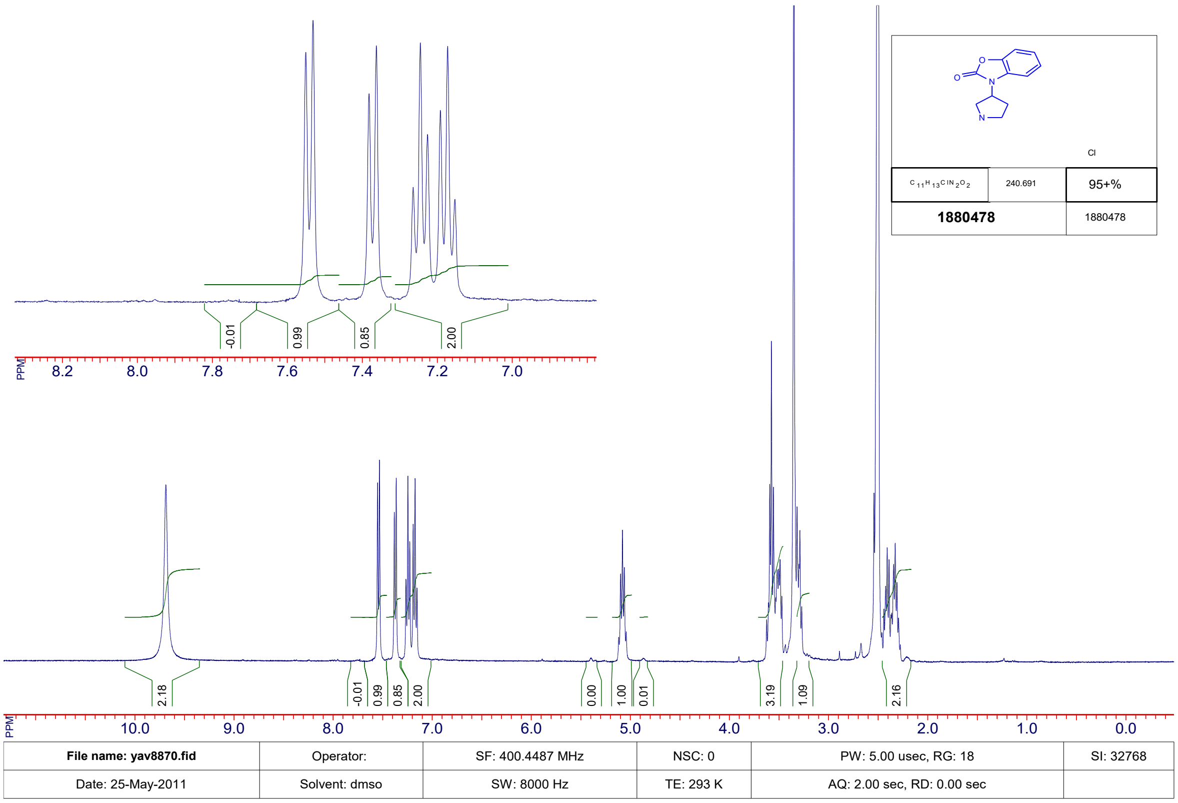
C11H13ClN2O2
240.691
95+%
1880478
1880478
File name: yav8870.fid
Operator:
SF: 400.4487 MHz
NSC: 0
PW: 5.00 usec, RG: 18
SI: 32768
Date: 25-May-2011
Solvent: dmso
SW: 8000 Hz
TE: 293 K
AQ: 2.00 sec, RD: 0.00 sec

F)

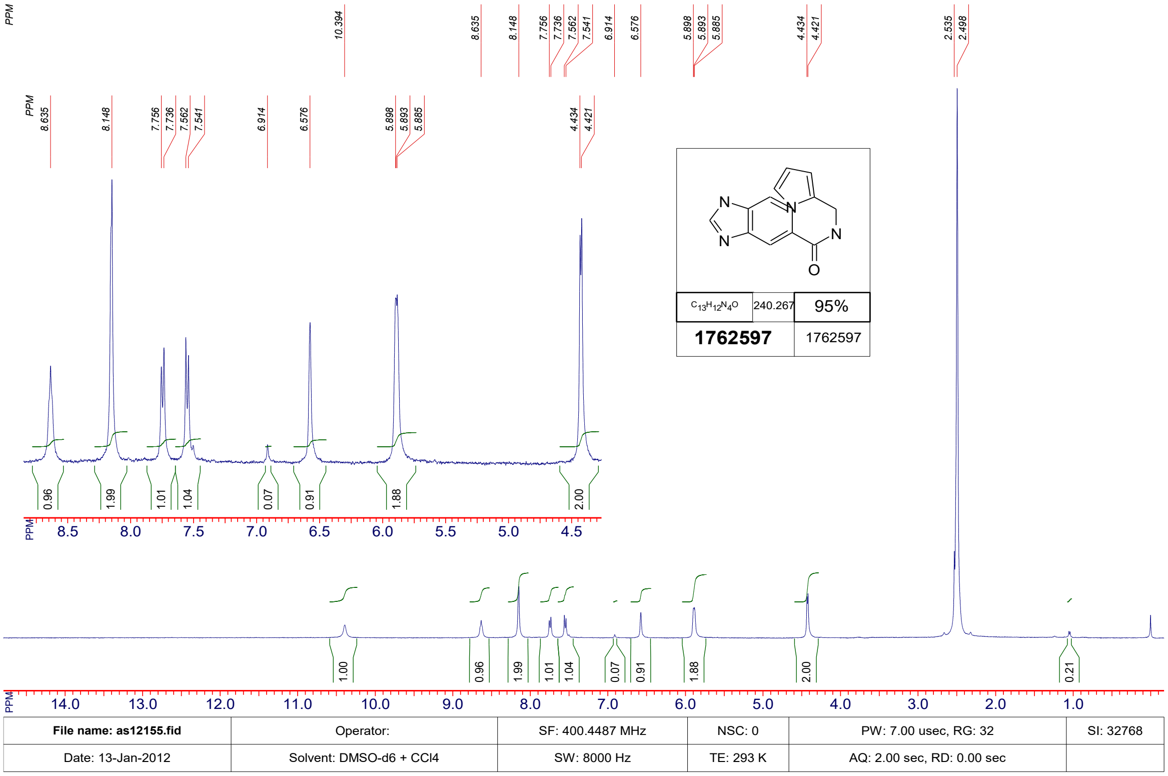
C13H12N4O
240.267
95%
1762597
1762597
File name: as12155.fid
Operator:
SF: 400.4487 MHz
NSC: 0
PW: 7.00 usec, RG: 32
SI: 32768
Date: 13-Jan-2012
Solvent: DMSO-d6 + CCl4
SW: 8000 Hz
TE: 293 K
AQ: 2.00 sec, RD: 0.00 sec

G)

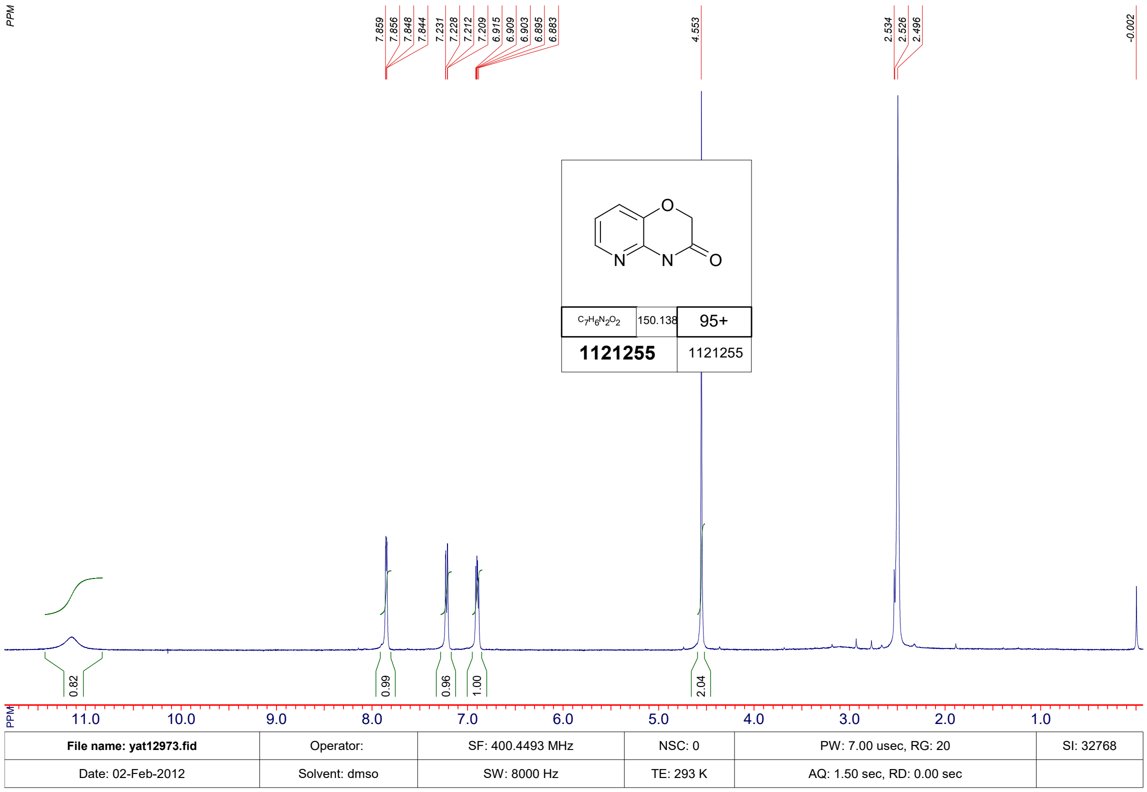

H)

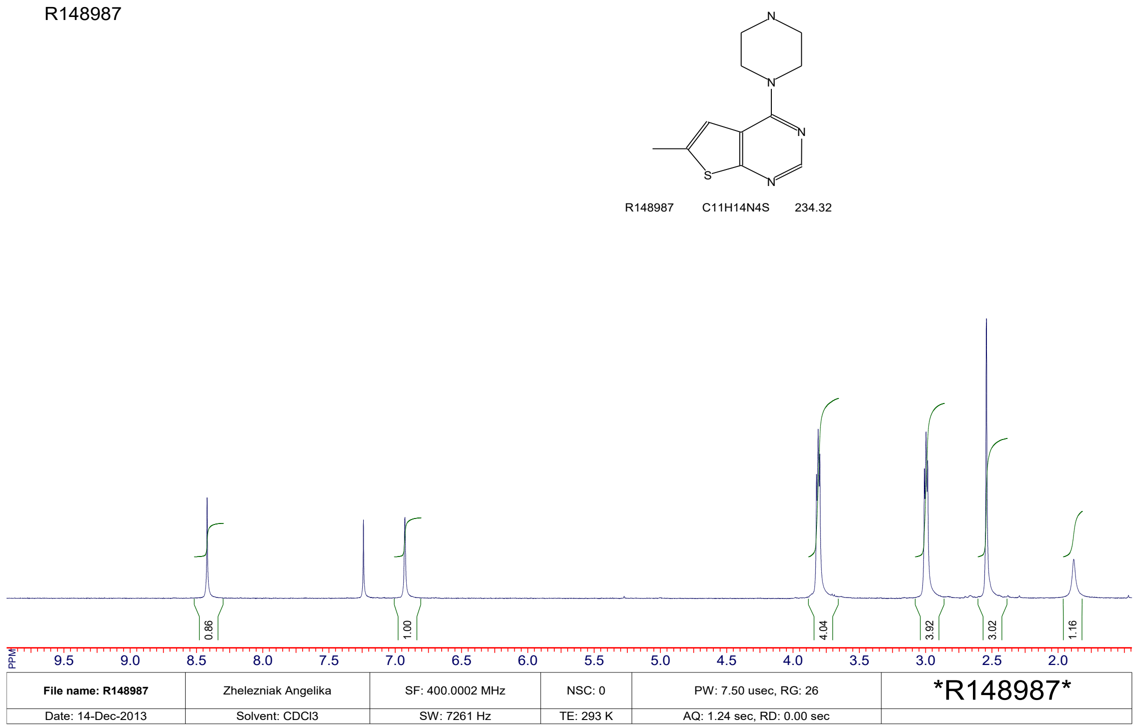

Fig. S6 Analytical data of confirmed binders. NMR data of A) **2**, B) **9**, C) **22**, D) **23**, E) **24**, F) **25**, G) **29**, and H) **31**.

A)

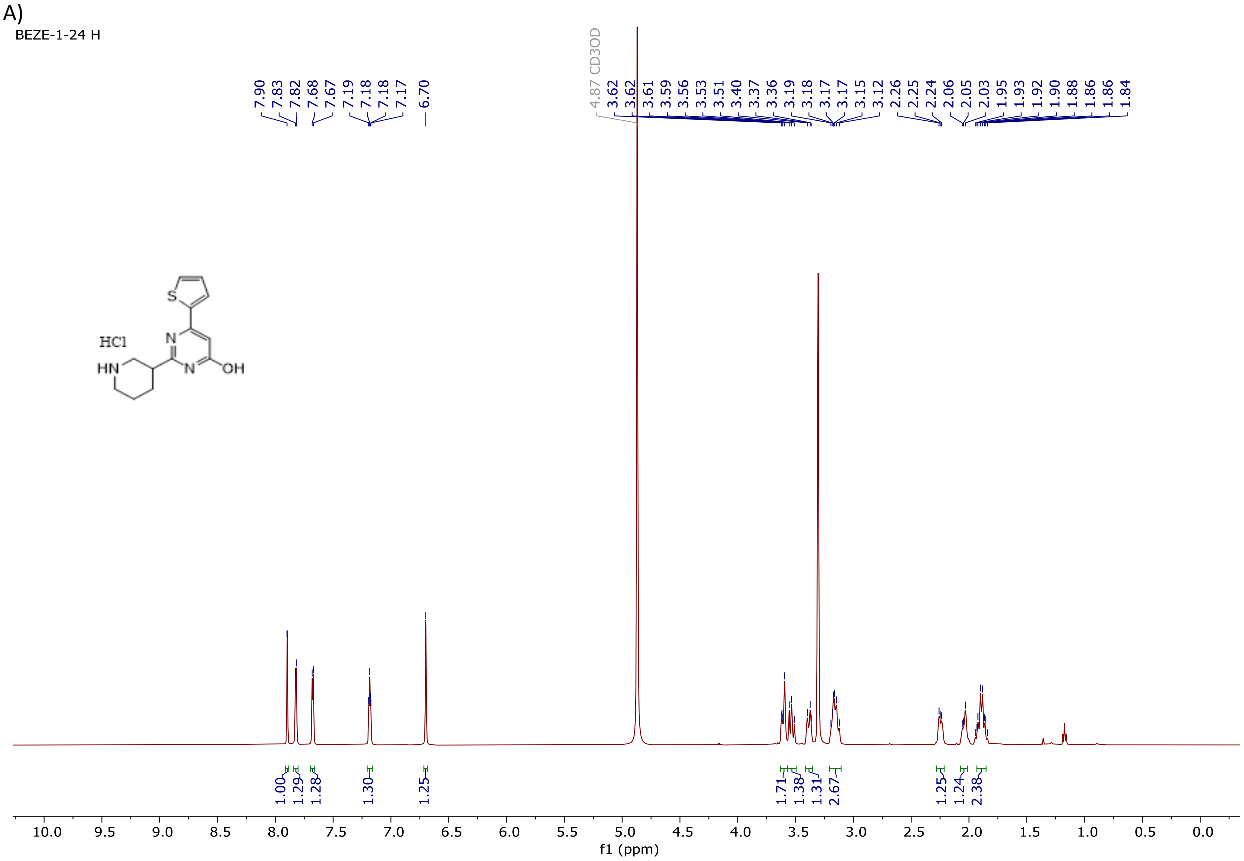
BEZE-1-24 H
HCl
HN
N
N
S
OH
7.90
7.83
7.82
7.68
7.67
7.19
7.18
7.18
7.17
6.70
4.87 CD3OD
3.62
3.62
3.61
3.59
3.56
3.53
3.51
3.40
3.37
3.36
3.19
3.18
3.17
3.17
3.15
3.12
2.26
2.25
2.24
2.06
2.05
2.03
1.95
1.93
1.92
1.90
1.88
1.86
1.86
1.84
1.00
1.29
1.28
1.30
1.25
1.71
1.38
1.31
2.67
1.25
1.24
2.38
f1 (ppm)

B)

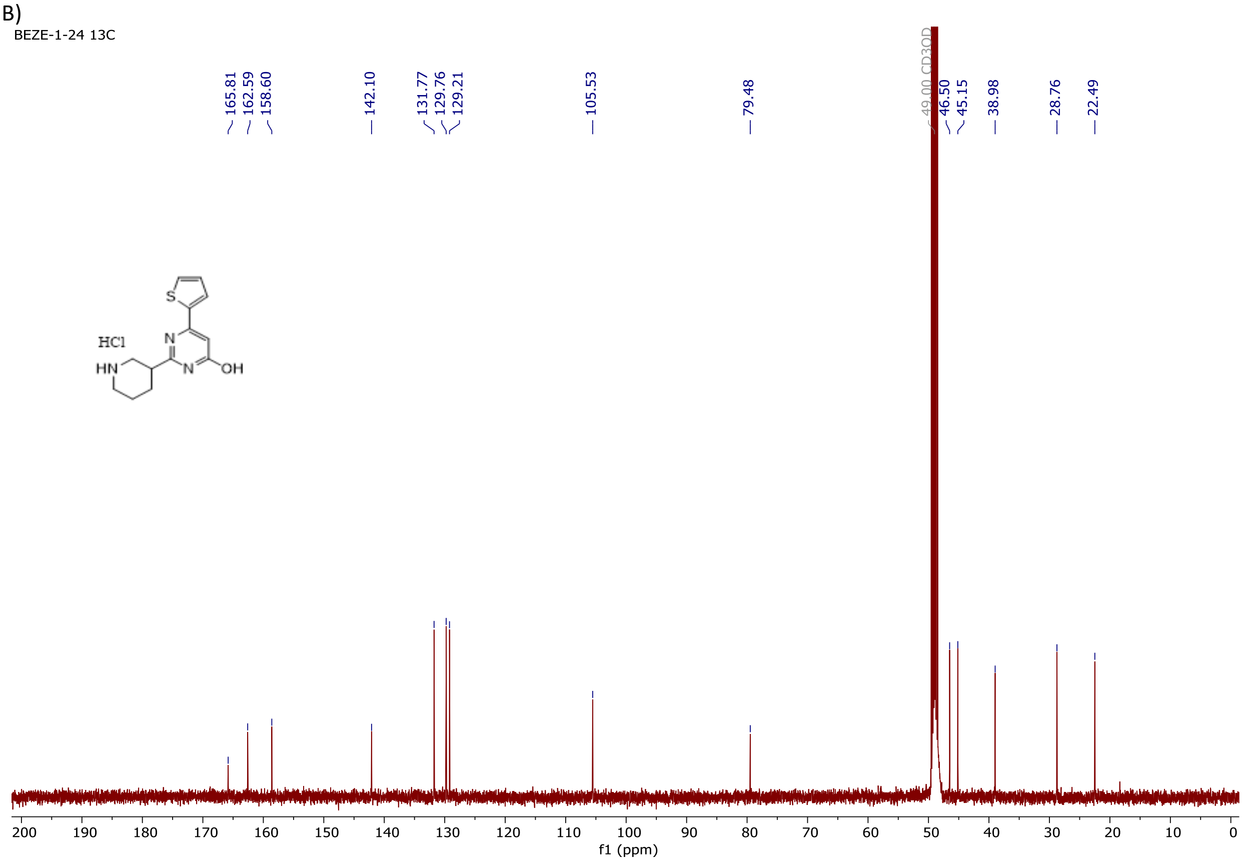
BEZE-1-24 13C
HCl
HN
N
N
S
OH
165.81
162.59
158.60
142.10
131.77
129.76
129.21
105.53
79.48
49.00 CD3OD
46.50
45.15
38.98
28.76
22.49
f1 (ppm)

C)

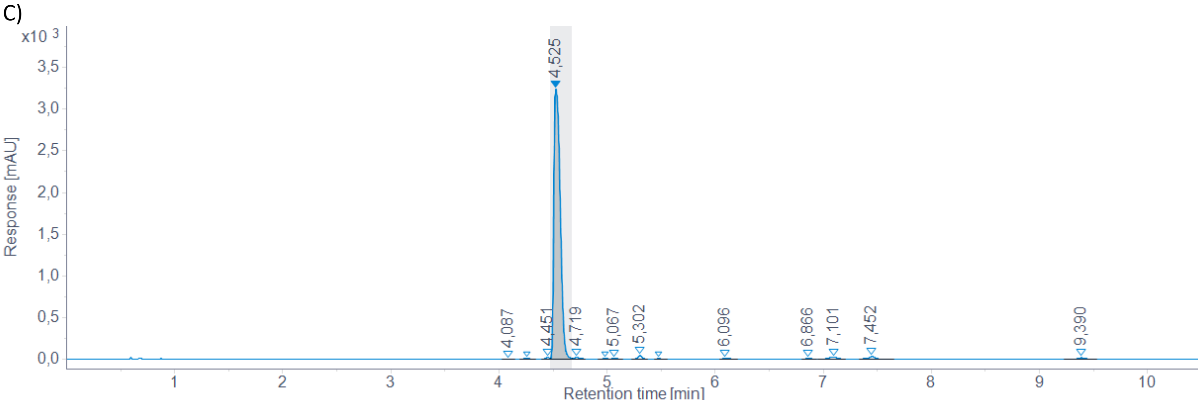

#	Name	Signal description	RT (min)	Area%	Area (mAU·s)	Height%
1		* DAD1A,Sig=254.0,10.0 Ref=off [b]	4,087	0,037	5,057	0,06
2		* DAD1A,Sig=254.0,10.0 Ref=off [b]	4,265	0,096	13,106	0,12
3		* DAD1A,Sig=254.0,10.0 Ref=off [b]	4,451	0,312	42,787	0,64
4		* DAD1A,Sig=254.0,10.0 Ref=off [b]	4,525	95,874	13155,058	94,75
5		* DAD1A,Sig=254.0,10.0 Ref=off [b]	4,719	0,422	57,888	0,47
6		* DAD1A,Sig=254.0,10.0 Ref=off [b]	4,987	0,108	14,757	0,17
7		* DAD1A,Sig=254.0,10.0 Ref=off [b]	5,067	0,150	20,605	0,24
8		* DAD1A,Sig=254.0,10.0 Ref=off [b]	5,302	0,703	96,465	1,14
9		* DAD1A,Sig=254.0,10.0 Ref=off [b]	5,479	0,061	8,359	0,08
10		* DAD1A,Sig=254.0,10.0 Ref=off [b]	6,096	0,181	24,868	0,18
11		* DAD1A,Sig=254.0,10.0 Ref=off [b]	6,866	0,095	13,072	0,11
12		* DAD1A,Sig=254.0,10.0 Ref=off [b]	7,101	0,765	104,927	0,67
13		* DAD1A,Sig=254.0,10.0 Ref=off [b]	7,452	0,809	110,971	0,93
14		* DAD1A,Sig=254.0,10.0 Ref=off [b]	9,390	0,388	53,283	0,44

Fig. S7 Analytical data of **1**. Charts above display A) ^{1}H-NMR spectrum B) ^{13}C-NMR and C) HPLC chromatogram of **1**.

Table S1: Structures and screening responses (in nm) of primary hits against the TPP and FMN riboswitches (RS), along with their classification, outcomes from dose-response assays using BLI, binding characteristics determined by NMR spectroscopy, and measured affinities for the FMN riboswitch.

Fragment	Structure	TPP RS	FMN RS	Category	Dose response against FMN RS	Dose response against TPP RS	NMR results	K_D [μM]
**2**	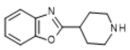	0.09	0.06	Common hit	Yes	absent	competitive	270
**3**	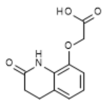	0.13	0.06	Common hit	Yes	absent	no binder	-
**4**	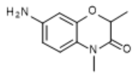	0.11	0.02	Common hit	< 5 data points	absent	not tested	-
**5**	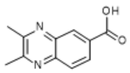	0.12	0.07	Common hit	< 5 data points	absent	not tested	-
**6**	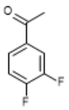	0.12	0.07	Common hit	Yes	absent	no binder	-
**7**	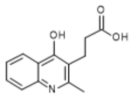	0.11	0.04	Common hit	< 5 data points	absent	not tested	-
**8**	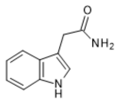	0.11	0.04	Common hit	Yes	absent	no binder	-
**9**	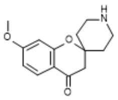	0.10	0.03	Common hit	Yes	absent	competitive	510
**10**	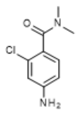	0.09	0.02	Common hit	Yes	absent	no binder	-
**11**	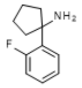	0.09	0.02	Common hit	Yes	absent	no binder	-
**12**	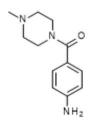	0.10	0.03	Common hit	Yes	absent	no binder	-
**13**	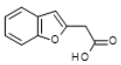	0.11	0.04	Common hit	Yes	absent	no binder	
**14**	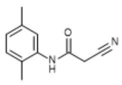	0.13	0.06	Common hit	Yes	absent	no binder	-
**15**	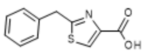	0.11	0.03	Common hit	Yes	absent	no binder	-

**16**	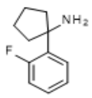	0.09	0.00	TPP RS hits	< 5 data points	absent	not tested	-
**17**	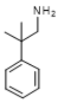	0.09	-0.01	TPP RS hits	< 5 data points	absent	not tested	-
**18**	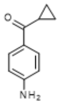	0.09	0.00	TPP RS hits	< 5 data points	absent	not tested	-
**19**	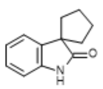	0.10	0.01	Common hit	< 5 data points	absent	not tested	-
**20**	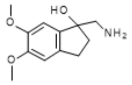	0.01	0.06	Common hit	Yes	absent	no binder	-
**21**	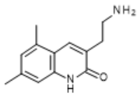	-0.01	0.01	FMN RS hits	No dose response	absent	not tested	-
**22**	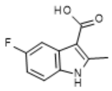	0.05	0.04	Common hit	Yes	absent	competitive	150
**23**	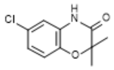	0.08	0.06	Common hit	Yes	absent	competitive	350
**24**	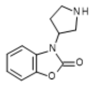	0.01	-0.01	TPP RS hits	Yes	absent	competitive	440
**25**	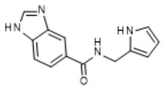	0.04	0.11	Common hit	Yes	absent	competitive	14
**26**	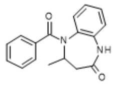	0.01	0.00	TPP RS hits	Yes	absent	no binder	-
**27**	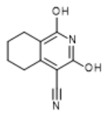	0.04	0.06	Common hit	No dose response	absent	not tested	-
**28**	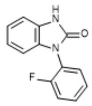	0.05	0.06	Common hit	< 5 data points	absent	not tested	-
**29**	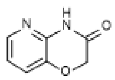	0.05	0.05	Common hit	Yes	absent	competitive	220
**30**	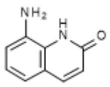	0.03	0.06	Common hit	absent	absent	not tested	-

**31**	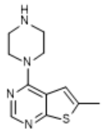	0.03	0.06	Common hit	Yes	Yes	Non-competitive	-
**32**	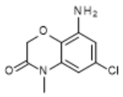	0.05	0.03	Common hit	absent	absent	not tested	-
**33**	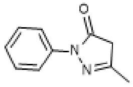	0.06	0.06	Common hit	Yes	absent	no binder	-
**34**	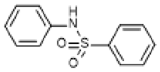	0.05	0.06	Common hit	Yes ($R^2 < 0.9$)	absent	no binder	-
**35**	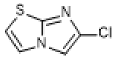	-0.01	0.01	FMN RS hits	< 5 data points	absent	not tested	-
**36**	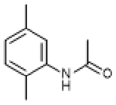	0.01	0.00	TPP RS hits	Yes	absent	no binder	-

Table S2 Nucleotide sequences of the riboswitches and their DNA templates used in this study. All sequences are presented in the 5' to 3' direction. "ssDNA" denotes single stranded synthetic *in vitro* transcription template with the region complementary to the T7 promoter consensus sequence underlined. "dsDNA" refers to double stranded *in vitro* transcription template, where only the top strand is shown, and the T7 promoter consensus sequence is underlined. The bold letter in the T7 promoter consensus sequence represents the first nucleotide of the transcript. The 2'-O'methylated nucleotides are indicated by placing "m" preceding the nucleotide.

**Element**	**Bacterial species**	**Backbone**	**Sequence**
FMN riboswitch	*Oscillibacter* sp. KLE 1745	ssDNA template	mGmATCTTCTTTCATCCAGACTTTACTGTCGGCCTTGGAGTTTCACCAAGTCTGCCCAGTTTCCTGGGCTCGCGGGCTGTACCGCCGGTGGGGAATTTCACCCCGCCCTGAAGAT**C**TATAGTGAGTCGTATTA
		RNA	GAUCUUCAGGGCGGGGUGAAAUUCCCCACCGGCGGUACAGCCCGCGAGCCCAGGAAACUGGGCAGACUUGGUGAAACUCCAAGGCCGACAGUAAAGUCUGGAUGAAAGAAGAUC
SAM-I riboswitch	*T. tengcongensis*	dsDNA template	TAATACGACTCACTATA**G**GCTTATCAAGAGAGGTGGAGGGACTGGCCCGACGAAACCCGGCAACCAGAAATGGTGCCAATTCCTGCAGCGGAAACGTTGAAAGATGAGCCG
		RNA	GGCUUAUCAAGAGAGGUGGAGGGACUGGCCCGACGAAACCCGGCAACCAGAAAUGGUGCCAAUUCCUGCAGCGGAAACGUUGAAAGAUGAGCCG
TPP riboswitch	*E. coli*	ssDNA template	mCmAGCACTTCCCTACGCTGGCATTATCCAGATCAGGTGATACGGGTATTTCTCAGCCTTCACGCAGAAGGGCACCCCGAGTACT**G**TATAGTGAGTCGTATTA
		RNA	CAGUACUCGGGGUGCCCUUCUGCGUGAAGGCUGAGAAAUACCCGUAUCACCUGAUCUGGAUAAUGCCAGCGUAGGGAAGUGCUG
T7 RNAP promotor		ssDNA template	TAATACGACTCACTATA**C**

Table S3 Similarity of known TPP riboswitch fragments with library compounds. Six compounds from the screening library have a Tanimoto similarity coefficient > 0.7 when compared to previously reported TPP riboswitch hits.[1,2]

Previously identified TPP riboswitch binder	Comment	Similar compounds in our library	Tanimoto coefficient
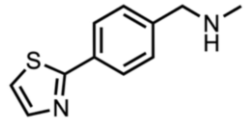	Competitive TPP riboswitch binder K_D = 325 μM (Cressina *et al.*)	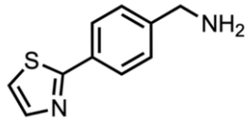 AA3B10	0.82
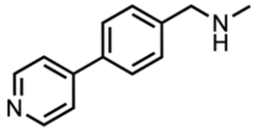	Nonspecific binder, showing binding against TPP and *lysC* riboswitch (Cressina *et al.*).	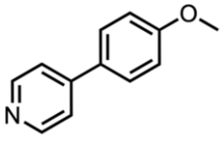 AA1E05	0.78
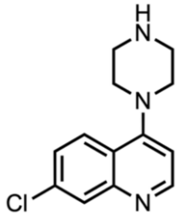	Nonspecific binder, showing binding against TPP and *lysC* riboswitch (Cressina *et al.*).	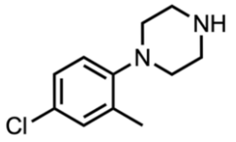 D1B04	0.73
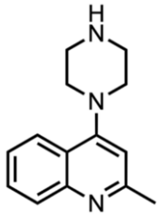	Insoluble (Meredith *et al.*) Nonspecific binder, showing binding against TPP and *lysC* riboswitch (Cressina *et al.*).	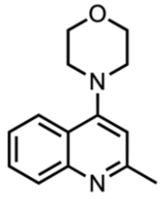 D1G09	0.82
		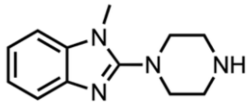 A1B06	0.72
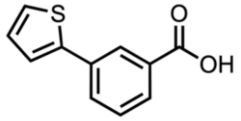	Insoluble (Meredith *et al.*)	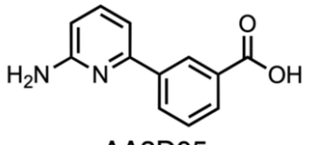 AA3D05	0.71

Chemistry

General experimental for synthesis part

Anhydrous ethanol was obtained from VWR and used as delivered. All other chemicals and solvents were purchased from Sigma-Aldrich and used as delivered unless otherwise stated. All moisture sensitive reactions were carried out under argon atmosphere in oven-dried (130 °C) equipment that has been cooled down under vacuum. Anhydrous THF was obtained from a sodium/benzophenone still or an anhydrous solvent delivery system (SPS-800 system from M. Braun GmbH, Garching, Germany). Flash column chromatography was performed using silica gel from Merck (Silica gel 60, 0.040 – 0.063 mm). Thin layer chromatography (TLC) analyses were performed on aluminum sheets coated with Merck TLC silica gel 60 F254 and visualization was achieved by using ultraviolet light (254 nm) or a solution of sodium permanganate. The NMR experiments were recorded on a Bruker BioSpin AV500. ^{1}H and ^{13}C chemical shifts (δ) are reported in ppm with reference to the solvent residual peak ($CDCl_3$: δH = 7.26 and δC = 77.16; DMSO-d_6: δH = 2.50 and δC = 39.98). All coupling constants are given in Hertz (Hz). HRMS-analyses were performed by Dr. Bjarte Holmelid on a AccuTOF™ JMS-T100LC from JEOL, USA, Inc. (Peabody, MA, USA) mass spectrometer that was operated with an orthogonal electrospray ionization source (ESI).

Synthesis scheme of 2-(Piperidin-3-yl)-6-(thiophen-2-yl)pyrimidin-4(3H)-one (**1**). The synthesis of compound **1** was carried out following a similar route as originally reported by Howe et. al.[3] Briefly, keto-ester S1 was prepared from Meldrum's acid and thiophene-2-carboxylic acid in excellent yield and was treated with NH_4OH at high temperature to afford 3-amino acrylamide S2. Reaction with Boc-protected methyl piperidine-3-carboxylate in the presence of excess NaOMe gave pyrimidone S3, which was deprotected to afford **1**.

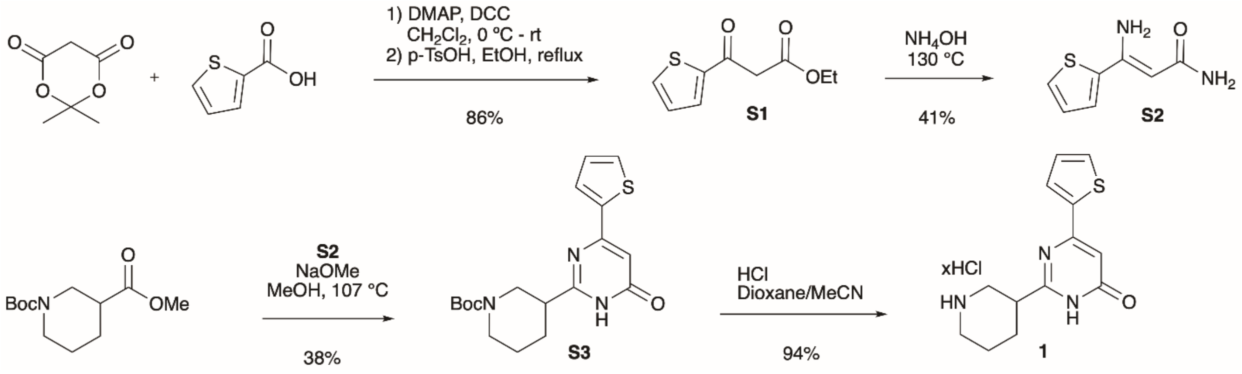

Scheme 1 Synthesis of ribocil fragment **1**.

Ethyl 3-oxo-3-(thiophen-2-yl)propanoate (**S1**)

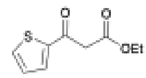

Thiophene-2-carboxylic acid (5.01 g, 39.1 mmol) and 2,2-dimethyl-1,3-dioxane-4,6-dione (6.75 g, 46.8 mmol) were dissolved in dry CH_2Cl_2 (125 mL) and cooled in an ice/water bath, before DMAP (9.51 g, 77.8 mmol) and DCC (8.88 g, 43.0 mmol) were added slowly. The resulting mixture was stirred at 0 °C for 20 min before the cooling bath was removed and stirring continued at room temperature for 3 h. The precipitated dicyclohexylurea was filtered off using a Büchner funnel, washed with CH_2Cl_2 and the filtrate was concentrated in vacuo. The residue was dissolved in anhydrous EtOH (250 mL) and a solution of p-TsOH (16.4 g, 95.1 mmol) in anhydrous EtOH (100 mL) was added slowly. The resulting mixture was heated to reflux for 1 h. After cooling to room temperature, the mixture was concentrated under reduced pressure, and the residue was dissolved in EtOAc (200 mL). The resulting solution was washed with water (200 mL), and the aqueous layer was extracted with EtOAc

(3 × 100 mL). The combined organic layers were washed with saturated $NaHCO_3$ (200 mL), 10% HCl (200 mL) and saturated NaCl (200 mL). The organic layer was dried over $MgSO_4$, filtered, and concentrated under reduced pressure. The crude product was purified via flash chromatography over silica gel using EtOAc/hexanes (1:9) as eluent yielding ethyl 3-oxo-3-(thiophen-2-yl)propanoate **S1** as an orange oil (6.66 g, 86%). R_f = 0.15 (EtOAc/hexanes, 1:9); ^{1}H-NMR (500 MHz, $CDCl_3$): δ = 7.73 (dd, J = 3.8, 1.1, 1H), 7.69 (dd, J = 4.9, 1.1, 1H), 7.13 (dd, J = 4.9, 3.8, 1H), 4.20 (q, J = 7.0, 2H), 3.90 (s, 2H), 1.25 (t, J = 7.0, 3H); ^{13}C-NMR (126 MHz, $CDCl_3$) δ = 185.3, 167.3, 143.6, 135.2, 133.6, 128.6, 61.9, 46.8, 14.4; HRMS (ESI): m/z calcd for $C_9H_{11}O_3S^+$ [M + H]$^+$: 199.0423; found: 199.0426.

3-Amino-3-(thiophen-2-yl)acrylamide (**S2**)

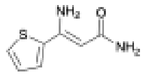

Keto ester **S1** (741 mg, 3.7 mmol) was dissolved 25% ammonium hydroxide (6 mL) in a 10 mL microwave vial which was sealed and heated at 130 °C for 3h. The reaction mixture was then placed in an ice bath for 20 minutes, and the solid material was isolated by filtration and washed with hexanes to yield 3-amino-3-(thiophen-2-yl)acrylamide **S2** as brown crystalline solid (254 mg, 41%). ^{1}H-NMR (500 MHz, CD_3OD): δ = 7.45 (d, J = 4.3, 2H), 7.08 (t, J = 4.3, 1H), 5.07 (s, 1H); ^{13}C-NMR (126 MHz, CD_3OD): δ = 175.3, 153.5, 142.0, 128.8, 128.0, 127.0, 85.7; HRMS (ESI): m/z calcd for $C_7H_9N_2OS^+$ [M + H]$^+$: 169.0430; found: 169.0438.

tert-Butyl 3-(6-oxo-4-(thiophen-2-yl)-1,6-dihydropyrimidin-2-yl)piperidine-1-carboxylate (**S3**)

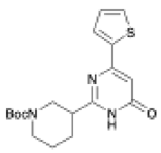

Elementary sodium (259 mg, 11.3 mmol) was dissolved in anhydrous MeOH (2.7 mL) in an oven dried 10 mL microwave vial and allowed to cool to room temperature before acryl amide **S2** (251 mg, 1.49 mmol) and 1-tert-butyl 3-ethyl piperidine-1,2-dicarboxylate (917 mg, 3.56 mmol) were added. The vial was sealed and heated at 108 °C for 22 h. The reaction mixture was then cooled in an ice/water bath before it was acidified to pH 5–6 by addition of 10% HCl and stirred at 0 °C for 30 min. The formed precipitate was filtered off, and the filtrate was extracted with EtOAc/THF (1:1, 3 × 50 mL). The organic layers were combined with the solid material and the resulting solution dried over $MgSO_4$, filtered and concentrated in vacuo. The crude product was purified via flash column chromatography over silica gel (EtOAc/CH_2Cl_2, 2:8, then EtOAc) yielding tert-butyl 3-(6-oxo-4-(thiophen-2-yl)-1,6-dihydropyrimidin-2-yl)piperidine-1-carboxylate **S3** as a pale-yellow solid material (206 mg, 38%). R_f = 0.37 (MeOH/EtOAc/hexanes, 1:3:6); ^{1}H-NMR (500 MHz, $CDCl_3$): δ = 7.68 (dd, J = 3.8, 1.2, 1H), 7.53 – 7.48 (m, 1H), 7.13 (dd, J = 5.0, 3.8, 1H), 6.62 (s, 1H), 4.25 (bs, 1H), 4.04 (bs, 1H), 3.30 (bs, 1H), 2.97 – 2.87 (m, 1H), 2.81 (ddd, J = 10.5, 6.6, 4.0, 1H), 2.20 – 2.12 (m, 1H), 1.94 – 1.80 (m, 2H), 1.71 – 1.57 (m, 1H), 1.45 (s, 9H); ^{13}C-NMR (126 MHz, $CDCl_3$): δ = 166.0, 163.1, 158.2, 155.1, 142.3, 130.6, 128.8, 127.8, 104.9, 80.2, 51.2, 47.3, 43.9, 41.8, 29.6, 28.8; HRMS (ESI): m/z calcd for $C_{18}H_{24}N_3O_3S^+$ [M + H]$^+$: 362.1533; found: 362.1540.

2-(Piperidin-3-yl)-6-(thiophen-2-yl)pyrimidin-4(3H)-one hydrochloride (1)

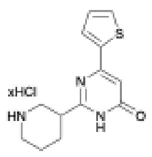

Boc-protected **S3** (212 mg, 0.59 mmol) was dissolved in a mixture of 1,4-diaoxane (22 mL) and acetonitrile (9 mL), after which HCl (4 M in 1,4-dioxane, 13.6 mL) was added and the reaction mixture was stirred at room temperature for 2.5 h.The reaction mixture was then concentrated in vacuo, and washed with diethyl ether, followed by cold CH_2Cl_2 yielding 2-(piperidin-3-yl)-6-(thiophen-2-yl)pyrimidin-4(3H)-one hydrochloride **1** as a colorless solid (164 mg, 94%). ^{1}H-NMR (500 MHz, CD_3OD): δ = 7.90 (s, 1H), 7.82 (d, J = 3.8, 1H), 7.68 (d, J = 4.9, 1H), 7.21 – 7.16 (m, 1H), 6.70 (s, 1H), 3.62-3.59 (m, 2H), 3.57 – 3.49 (m, 1H), 3.40-3.36 (m, 1H), 3.21 – 3.11 (m, 3H), 2.28 – 2.21 (m, 1H), 2.06-2.03 (m, 1H), 1.95-1.84 (m, 2H); ^{13}C-NMR (126 MHz, CD_3OD): δ = 165.8, 162.6, 158.6, 142.1, 131.8, 129.8, 129.2, 105.5, 79.5, 46.5, 45.2, 39.0, 28.8, 22.5; HRMS (ESI): m/z calcd for $C_{13}H_{16}N_3OS^+$ [M + H]$^+$: 262.1009; found: 262.1016.

References

1 E. Cressina, L. Chen, C. Abell, F. J. Leeper and A. G. Smith, Fragment screening against the thiamine pyrophosphate riboswitch thiM, *Chemical Science*, 2011, **2**, 157–165.

2 M. J. Zeller, O. Favorov, K. Li, A. Nuthanakanti, D. Hussein, A. Michaud, D. A. Lafontaine, S. Busan, A. Serganov, J. Aubé and K. M. Weeks, SHAPE-enabled fragment-based ligand discovery for RNA, *Proc. Natl. Acad. Sci. U. S. A.*, 2022, **119**, e2122660119.

3 J. A. Howe, H. Wang, T. O. Fischmann, C. J. Balibar, L. Xiao, A. M. Galgoci, J. C. Malinverni, T. Mayhood, A. Villafania, A. Nahvi, N. Murgolo, C. M. Barbier, P. A. Mann, D. Carr, E. Xia, P. Zuck, D. Riley, R. E.Painter, S. S. Walker, B. Sherborne, R. de Jesus, W. Pan, M. A. Plotkin, J. Wu, D. Rindgen, J. Cummings, C. G. Garlisi, R. Zhang, P. R. Sheth, C. J. Gill, and Haifeng Tang & Terry Roemer, Selective small-molecule inhibition of an RNA structural element, *Nature*, 2015, **526**, 672–677.
